# *Lepotrema* Ozaki, 1932 (Lepocreadiidae: Digenea) from Indo-Pacific fishes, with the description of eight new species, characterised by morphometric and molecular features

**DOI:** 10.1007/s11230-018-9821-1

**Published:** 2018-10-15

**Authors:** Rodney A. Bray, Scott C. Cutmore, Thomas H. Cribb

**Affiliations:** 1 Department of Life Sciences, Natural History Museum, Cromwell Road, London, SW7 5BD UK; 2 School of Biological Sciences, The University of Queensland, St Lucia, QLD 4072 Australia

## Abstract

We review species of the genus *Lepotrema* Ozaki, 1932 from marine fishes in the Indo-West Pacific. Prior to the present study six species were recognised. Here we propose eight new species on the basis of combined morphological and molecular analysis: *Lepotrema acanthochromidis* n. sp. ex *Acanthochromis polyacanthus* from the Great Barrier Reef (GBR); *Lepotrema hemitaurichthydis* n. sp. ex *Hemitaurichthys polylepis* and *H. thompsoni* from Palau and French Polynesia; *Lepotrema melichthydis* n. sp. ex *Melichthys vidua* from Palau and the GBR; *Lepotrema amansis* n. sp. ex *Amanses scopas* from the GBR; *Lepotrema cirripectis* n. sp. ex *Cirripectes filamentosus*, *C. chelomatus* and *C. stigmaticus* from the GBR; *Lepotrema justinei* n. sp. ex *Sufflamen fraenatum* from New Caledonia; *Lepotrema moretonense* n. sp. ex *Prionurus microlepidotus*, *P. maculatus* and *Selenotoca multifasciata* from Moreton Bay; and *Lepotrema amblyglyphidodonis* n. sp. ex *Amblyglyphidodon curacao* and *Amphipron akyndynos* from the GBR. We also report new host records and provide novel molecular data for two known species: *Lepotrema* *adlardi* Bray, Cribb & Barker, 1993 and *Lepotrema* *monile* Bray & Cribb, 1998. Two new combinations are formed, *Lepotrema cylindricum* (Wang, 1989) n. comb. (for *Preptetos cylindricus*) and *Lepotrema navodonis* (Shen, 1986) n. comb. (for *Lepocreadium navodoni*). With the exception of a handful of ambiguous records, the evidence is compelling that the host-specificity of species in this genus is overwhelmingly oioxenous or stenoxenous. This renders the host distribution in three orders and ten families especially difficult to explain as many seemingly suitable hosts are not infected. Multi-loci molecular data (ITS2 rDNA, 28S rDNA and *cox*1 mtDNA) demonstrate that *Lepotrema* is a good generic concept, but limited variability in sequence data and differences in phylogenies produced for different gene regions make relationships within the genus difficult to define.

## Introduction

Members of the digenean family Lepocreadiidae Odhner, 1905 are common parasites of fishes of the Indo-West Pacific region, particularly of coral reef fishes. Our systematic studies on this family in the waters around northern Australia and other sites in the region have hitherto been mostly reliant on comparative morphology (Barker et al., 1993; Bray et al., 1993; Bray & Cribb, 1996a, b, c, d; Bray et al., 1996a, b; Bray & Cribb, 1998; Bray et al., 1998; Bray & Nahhas, 1998; Bray & Cribb, 2002, 2003; Bray & Justine, 2006; Bray et al., 2009a, b; Bray et al., 2010a, b; Bray & Justine, 2012). More recently, some progress has been made in our understanding of higher level lepocreadiid systematics (Bray et al., 2009b; Bray & Cribb, 2012; Bray et al., 2018) by the addition of molecular evidence. At the species level, the limits of the discriminating ability of morphological evidence has become increasingly apparent as we have attempted to elucidate the systematics of some of the larger lepocreadiid genera. *Lepotrema* Ozaki, 1932 is a case in point. It is encountered in a wide range of fish families in the orders Tetraodontiformes and Perciformes, with one record from a pleuronectiform. Most of the literature reports, including some from all three orders, are listed under the type-species, *Lepotrema clavatum* Ozaki, 1932, suggesting a very low level of specificity. Our molecular evidence presented here, based mainly on ITS2 rDNA and *cox*1 mtDNA sequences, together with sampling evidence, however, indicates that in general specificity is high. Most species appear to be oioxenic or stenoxenic, with no clear evidence that any individual species parasitizes multiple orders.

In addition to the molecular and host-specificity evidence presented here, we have found that it is usually possible to detect minor, but relatively consistent, morphometric distinguishing characteristics if the sample from a given host is of a reasonable size, i.e. more than three specimens. Members of *Lepotrema* are small, making the use of hologenophores problematical, as most distinguishing characters are ratios of measurements relative to body-length. Nevertheless, most species are recognisable by combinations of morphometric characters, most readily visualised using graphs. In several cases, only one or two worms were recovered from a host species, and we have not been able to identify them to species.

## Materials and methods

Digeneans collected from freshly killed fish were fixed by being pipetted into nearly boiling saline and immediately preserved in formalin or 70% ethanol (Cribb & Bray, 2010). Whole-mounts were stained with Mayer’s paracarmine or Mayer’s haematoxylin, cleared in beechwood creosote or methyl salicylate and mounted in Canada balsam. Measurements were made through a drawing tube on an Olympus BH-2 microscope, using a Digicad Plus digitising tablet and Carl Zeiss KS100 software adapted by Imaging Associates, and are quoted in micrometres, with the range and the mean in parentheses. Morphometric distinctions are derived from graphs produced using the Scatter plot function in Excel. The following abbreviations are used: NHMUK, the Natural History Museum, London, UK; MNHN JNC, Muséum National d’Histoire Naturelle, Paris, France; QM, Queensland Museum, Brisbane, Australia; WAM, Western Australian Museum, Perth, Western Australia.

Specimens for molecular analysis were processed according to the protocols used by Sun et al. (2014) and Wee et al. (2017). The complete ITS2 rDNA region was amplified and sequenced using the primers 3S (Morgan & Blair, 1995) and ITS2.2 (Cribb et al., 1998), the partial D1-D3 28S rDNA region using LSU5 (Littlewood, 1994), 300F (Littlewood et al., 2000), ECD2 (Littlewood et al., 1997) and 1500R (Snyder & Tkach, 2001) and the partial *cox*1 mtDNA region using Dig_cox1Fa (Wee et al., 2017) and Dig_cox1R (Wee et al., 2017). Geneious® version 10.2.3 (Kearse et al., 2012) was used to assemble and edit contiguous sequences and the start and end of the ITS2 rDNA region were determined by annotation through the ITS2 Database (Keller et al., 2009; Ankenbrand et al., 2015) using the ‘Metazoa’ model.

ITS2 rDNA and *cox*1 mtDNA sequence data generated during this study were aligned in MEGA version 6 (Tamura et al., 2013), using MUSCLE version 3.7 (Edgar, 2004) with UPGMB clustering for clustering for iterations 1 and 2. Differences between taxa were displayed by performing an unrooted Neighbour-joining analysis on each dataset using the following conditions: “model/method = No. of differences”, “Substitutions to include = d: Transitions + Transversions” and “Gaps/Missing Data Treatment = complete deletion”. Nodal support was estimated by performing 10,000 bootstrap replications. Pairwise differences were estimated for each dataset using the following conditions: “variance estimation method = none”, “model/method = No. of differences” and “Substitutions to include = d: Transitions + Transversions” and “Gaps/Missing Data Treatment = complete deletion”. Species delineation was tested using the Automatic Barcode Gap Discovery (ABGD) method (Puillandre et al., 2012) to assign candidate species from the aligned *cox*1 mtDNA dataset; analysis was conducted using the ABGD web tool with the following parameters: “Pmin = 0.001”, “Pmax = 0.01”, “steps = 10”, “X (relative gap width) = 1.5”, “Nb bins = 20” and “distance = Jukes-Cantor”.

The partial 28S rDNA sequences generated during this study were aligned with sequences of related lepocreadiids from GenBank using MUSCLE version 3.7 (Edgar, 2004) run on the CIPRES portal (Miller et al., 2010a), with ClustalW sequence weighting and UPGMA clustering for iterations 1 and 2. The resultant alignment was refined by eye using MESQUITE (Maddison & Maddison, 2018). The ends of each sequence were trimmed and ambiguously aligned regions were identified and masked manually (those constituting more than three bases and present in greater than 5% of the sequences in the dataset). Bayesian inference analysis of the 28S dataset was performed using MrBayes version 3.2.6 (Ronquist et al., 2012), run on the CIPRES portal. The best nucleotide substitution model was estimated using jModelTest version 2.1.10 (Darriba et al., 2012); the TVM+I+Γ model was predicted the as the best estimator by the Akaike Information Criterion (AIC) and TPM2uf+I by the Bayesian Information Criterion (BIC). Bayesian inference analysis was run over 10,000,000 generations (ngen = 10,000,000) with two runs each containing four simultaneous Markov Chain Monte Carlo (MCMC) chains (nchains = 4) and every 1,000th tree saved. Bayesian inference analysis used the following parameters: “nst = 6”, “rates = invgamma”, “ngammacat = 4”, and the priors parameters of the combined dataset were set to “ratepr = variable”. Samples of substitution model parameters, and tree and branch lengths were summarised using the parameters “sump burnin = 3,000” and “sumt burnin = 3,000”. Species of *Mobahincia* Bray, Cribb & Cutmore, 2018 (MH157068), *Multitestis* Manter, 1931 (MH157071) and *Neomultitestis* Machida, 1982 (MH157072) were designated as functional outgroup taxa, following Bray et al. (2018).

## Results

**Family Lepocreadiidae Odhner, 1905**

**Genus** ***Lepotrema*** **Ozaki, 1932**

The genus *Lepotrema* was erected by Ozaki (1932) for *L. clavatum* Ozaki, 1932 from a monacanthid, the threadsail filefish *Stephanolepis cirrhifer* (Temminck & Schlegel) (as *Monacanthus c.*), from the Japanese coast. The exact site of collection was not given, but this fish species is said to be distributed between Otaru and Nagasaki. A “genital sucker” is described “at the bottom of the chamber (genital atrium) lying directly inside of the end part of the metraterm”. This feature, along with the distinctly dorsal excretory pore, represent the main distinguishing features of the genus. Yamaguti (1934) recognised the genus and species and reported it from the type-host in the Inland Sea, Japan, as well as in the Korean black scraper *Thamnaconus modestus* (Günther) (as *Cantherhines unicornu*) (Monacanthidae) and the cinnamon flounder *Pseudorhombus cinnamoneus* (Temminck & Schlegel) (Paralichthyidae) from Japanese waters. Some measurements were given but no illustration. The morphological features of the genus were not discussed. Four years later, Yamaguti (1938) re-examined the specimens from *S. cirrhifer* and *T. modestus* and synonymised the genus with *Lepocreadium* Stossich, 1903. He stated “Although Ozaki distinguished his genus *Lepotrema* from the known members of the Lepocreadiinae by the position of the genital pore and the possession of a genital sucker, the genital pore usually lies to one side of the median line in this subfamily as defined by Odhner and the “genital sucker” of Ozaki is not a sucker in the true sense of the word, but a bulb-like muscular thickening of the metraterm”. Hanson (1955) followed this generic designation in reporting (but not illustrating) *Lepocreadium clavatum* in ‘*Melichthys buniva*’, apparently a misapplied name for the black triggerfish *Melichthys niger* (Bloch) (Balistidae) (see Randall, 2007), from Hawaii. She also described *Lepocreadium incisum* Hanson, 1955 from the same fish species, reporting a “bulb-like muscular thickening of metraterm as described by Yamaguti” and a “subterminal” excretory pore. Pritchard (1963) reported *Lepocreadium clavatum* from *M. niger*, the pinktail triggerfish *Melichthys vidua* (Richardson), the brown-and-white butterflyfish *Hemitaurichthys zoster* (Bennett) (Chaetodontidae) and the Hawaiian dascyllus *Dascyllus albisella* Gill (Pomacentridae), all from Hawaii, but again without any illustration. Yamaguti (1970) re-recorded *Lepocreadium clavatum* from *M. vidua* from off Hawaii, describing it in detail, but illustrating only the terminal genitalia and the proximal female system. The terminal part of the metraterm is described as an indistinct “bipartite spherical bulb of lamellar muscle fibers”. He described a new species, *Lepocreadium xanthichthydis* Yamaguti, 1970 from the sargassum triggerfish *Xanthichthys ringens* (Linnaeus) (Balistidae) from off Hawaii with the metraterm provided with a “bulb of lamellar muscle fibers”, and a “dorsoterminal” excretory pore. Dyer et al. (1988) reported *Lepocreadium clavatum* in the white-banded triggerfish *Rhinecanthus aculeatus* (Linnaeus) (Balistidae) off Okinawa, Japan, but again without any illustration. Bray et al. (1993) reported *Lepocreadium clavatum* from two pomacentrids, the spiny chromis *Acanthochromis polyacanthus* (Bleeker) and the banded parma *Parma polylepis* Günther, from off Heron Island on the southern Great Barrier Reef, providing illustrations of individuals from both fishes. The terminal part of the metraterm was decribed as a “large, circular, folded muscular pad (not a sphincter)” and the excretory pore as “mid-dorsal, about halfway between caecal ends and posterior extremity”. They also decribed *Lepocreadium adlardi* Bray, Cribb & Barker, 1993 from the Bengal sergeant *Abudefduf bengalensis* (Bloch) (Pomacentridae), with the distal extremity of the metraterm “clamped in prominent folded muscular pad” and the excretory pore “mid-dorsal, close to level of posterior extremity of caeca”.

Bray et al. (1993) first considered re-recognising the genus *Lepotrema* based on the structure of the distal metraterm and the dorsal excretory pore. This action was then taken by Bray & Cribb (1996c) in a review of the genus. They transferred *Lepocreadium incisum*, *Lepocreadium xanthichthydis* and *Lepocreadium adlardi* to *Lepotrema* making new combinations. They also noted the similarity of *Preptetos cylindricus* Wang, 1989 and *Lepocreadium navodoni* Shen, 1986 to members of the genus, but refrained from making new combinations “pending further study”. They reported *Lepotrema clavatum* from the broom filefish *Amanses scopas* (Cuvier) (Monacanthidae) (illustrated) and the halfmoon triggerfish *Sufflamen chrysopterum* (Bloch & Schneider) (Balistidae) (not illustrated) from off Heron Island and described *Lepotrema canthescheniae* Bray & Cribb, 1996 from the endemic large-scaled leatherjacket *Cantheschenia grandisquamis* Hutchins from off Heron Island. Bray & Cribb (1998) erected *Lepotrema monile* Bray & Cribb, 1998 from Ward’s damsel *Pomacentrus wardi* Whitley (Pomacentridae) from off Heron Island. This species is problematical in that the distal metraterm is surrounded only by a “distinct, but narrow, sphincter”. The excretory pore, however, is “dorsal, between ends of caeca”. Machida & Kuramochi (1999) recognised the validity of *Lepotrema* in reporting (but not illustrating) *L. clavatum* in *T. modestus* and the red-toothed triggerfish *Odonus niger* (Rüppell) (Balistidae) off Japan. Machida & Uchida (2001) made the first report of *L. clavatum* from a pomacanthid when they recorded it from the Japanese swallow *Genicanthus semifasciatus* (Kamohara) from off Japan. They gave some measurements but did not describe the metraterm or excretory pore or give an illustration. In his review of the family Bray (2005) recognised the genus and Bray et al. (2009b) included ‘*L. clavatum*’ from *Acanthochromis polyacanthus* from off Lizard Island on the northern Great Barrier Reef in a molecular phylogeny of the Lepocreadioidea. The 28S rDNA and mitochondrial NADH dehydrogenase subunit 1 (ND1) reported by Bray et al. (2009b) for ‘*Lepotrema clavatum*’ are the only molecular data presently available for this genus.

The only evidence of the life-cycle of *Lepotrema* is supplied by Kondo et al. (2016), who described metacercariae of *L. clavatum* from three cnidarians, the moon jellyfish *Aurelia aurita* (Linnaeus) (*s.l*.), the Japanese sea nettle *Chrysaora pacifica* (Goette) and the ghost jellyfish *Cyanea nozakii* Kishinouye, from the Seto Inland Sea, Japan. They also reported metacercariae and juveniles of *L. clavatum* from juvenile Pacific rudderfish *Psenopsis anomala* (Temminck & Schlegel) (Centrolophidae) and *Thamnaconus modestus*. The first intermediate hosts are unknown.

**Overview of new findings**

In the present study we examined new specimens consistent with the concept of *Lepotrema* in the possession (especially) of a distinct folded muscular bulb on the distal metraterm and a postero-dorsal excretory pore. These were from 29 host/parasite/locality combinations. These forms are superficially highly similar to each other. The specimens were therefore assessed iteratively by morphology and analysis of ITS2 rDNA and *cox*1 mtDNA sequences for as many combinations for which suitable specimens were available. As discussed in greater detail below, we found that for host/locality combinations for which there were multiple specimens and multiple sequences, there was a strong tendency for genetic and morphological distinctions to be detectable. Using this rationale, we here characterise nine species on the basis of combined morphological and molecular data; of these seven are described as new. One further species is described as new on the basis of morphological data only. In addition, six existing species for which no molecular data are available are recognised. Finally, we summarise reports, old and new, of five host/parasite combinations which may well comprise further new species but for which the evidence is presently inadequate.

**Molecular data**

**Fig. 1:**
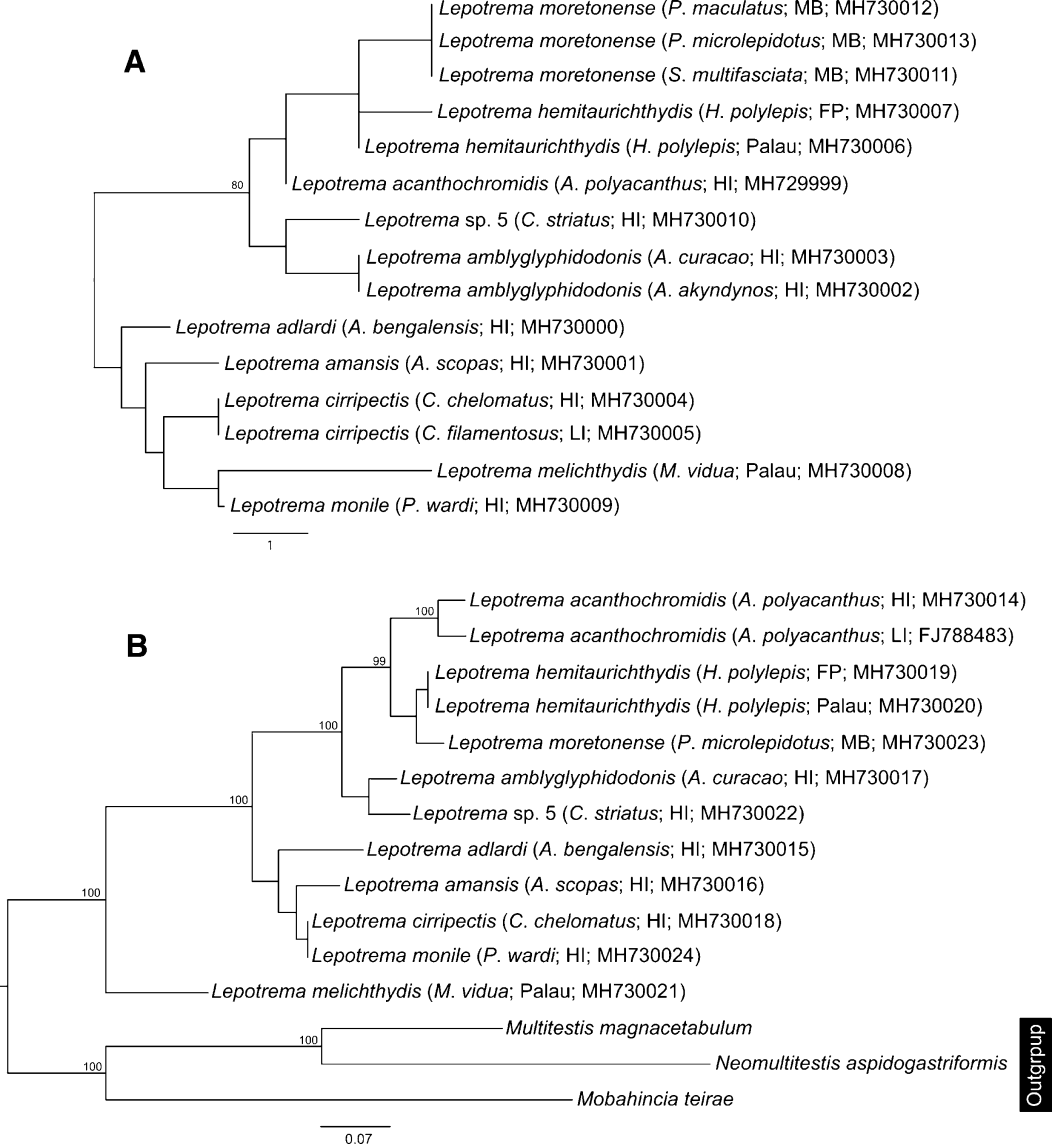
Phylogenetic analyses of the *Lepotrema* rDNA datasets. A, Phylogram from the unrooted Neighbour-joining analysis of the 5.8S-ITS2-28S dataset. Bootstrap support values shown at the nodes, with values of < 85 not shown. The scale-bar indicates the number of base differences; B, Phylogram from the Bayesian inference analysis of the 28S dataset. Posterior probabilities shown at the nodes, with values of < 85 not shown. *Abbreviations*: MB, Moreton Bay; FP, Austral Islands, French Polynesia; HI, Heron Island; LI, Lizard Island

**Fig. 2:**
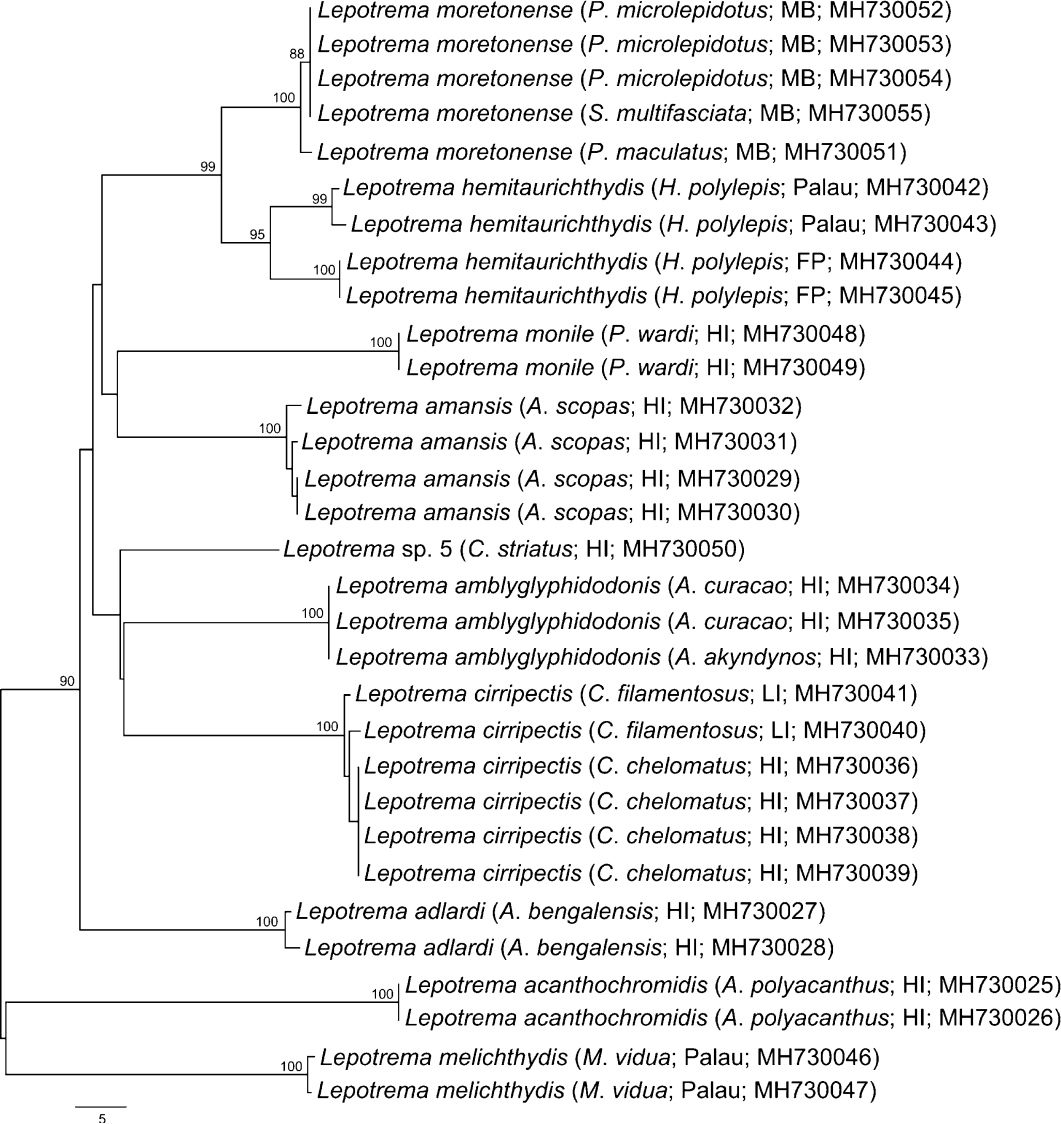
Phylogram from the unrooted Neighbour-joining analysis of the *cox*1 mtDNA dataset. Bootstrap support values shown at the nodes, with values of < 85 not shown. *Abbreviations*: MB, Moreton Bay; FP, Austral Islands, French Polynesia; HI, Heron Island; LI, Lizard Island

We generated 32 5.8S-ITS2-28S rDNA sequences and 31 partial *cox*1 mtDNA sequences for 15 host/locality combinations. Eleven genotypes/clades were present in both the ITS2 and *cox*1 datasets, several of which exhibited some low-level intra-genotypic variation. The complete ITS2 region ranged between 275–293 bp in length (as calculated by the ITS2 Database). The ITS2 alignment (including flanking 5.8S and 28S regions) comprised 11 genotypes (each represented by 1–6 replicates) and consisted of 466 bp. The level of distinction between ITS2 genotypes in the final dataset ranged from 1–9 bp (see Table 1); a phylogram representing these differences is shown in Fig. 1A. All *cox*1 sequences were 475 bp long and the final alignment contained no indels. The number of base differences between species ranged between 19–84 bp, and within a species between 0–14 bp (Table 1). A phylogram representing these differences is shown in Fig. 2. A striking aspect of this dataset is that genotypes in the ITS2 dataset differed by very few bases; several of the genotypes (ultimately considered to relate to different species) differed by just one base. However, in the *cox*1 dataset the same clades were represented with much greater levels of difference between them. Most of the ITS2 genotypes and *cox*1 clades related to single host/locality combinations. ABGD analysis of the *cox*1 dataset suggested the presence of 9–11 species. The initial partition identified nine groups; these nine groups matched the species recognised by morphology, except for the grouping of specimens from Palau, French Polynesia and Moreton Bay as a single unit. The recursive partition identified 11 groups; these groups matched the species recognised by morphology, except for the division of samples from Palau and French Polynesia. 28S rDNA sequence data were generated for all 11 genotypes and were 1,343–1,344 bp long; the final dataset (including the only *Lepotrema* sequence data available on GenBank and outgroup taxa) was 1,339 bp long. The level of differences between species ranged between 0–23 bp; two genotypes that had no bases different in the final dataset (those from *P*. *wardi* and those from *Cirripectes* spp.) differed by a single indel only. A phylogram representing analyses of the 28S dataset is shown in Fig. 1B.

**Table 1:** Total pairwise differences between *Lepotrema* species, with *cox*1 mtDNA sequences below and 5.8S-ITS2-28S rDNA sequences above the diagonal. Each host/parasite/locality/genotype combination for each gene region is represented by a single sequence

*Lepotrema* species	1	2	3	4	5	6	7	8	9	10	11	12	13	14	15	16	17	18	19	20	21
1. *L*. *moretonense* MB (MH730054)	–	0	0	–	1	2	6	–	6	–	4	4	4	6	–	6	6	–	2	–	9
2. *L*. *moretonense* MB (MH730055)	0	–	0	–	1	2	6	–	6	–	4	4	4	6	–	6	6	–	2	–	9
3. *L*. *moretonense* MB (MH730051)	2	2	–	–	1	2	6	–	6	–	4	4	4	6	–	6	6	–	2	–	9
4. *L*. *hemitaurichthydis* Palau (MH730042)	20	20	19	–	–	–	–	–	–	–	–	–	–	–	–	–	–	–	–	–	–
5. *L*. *hemitaurichthydis* Palau (MH730043)	22	22	21	2	–	1	5	–	5	–	3	3	3	5	–	5	5	–	1	–	8
6. *L*. *hemitaurichthydis* FP (MH730044)	21	21	21	14	14	–	6	–	6	–	4	4	4	6	–	6	6	–	2	–	9
7. *L*. *monile* HI (MH730048)	49	49	49	53	52	53	–	–	2	–	6	6	6	2	–	2	2	–	4	–	–
8. *L*. *amansis* HI (MH730032)	42	42	42	47	45	43	46	–	–	–	–	–	–	–	–	–	–	–	–	–	–
9. *L*. *amansis* HI (MH730031)	42	42	42	45	43	41	46	3	–	–	6	6	6	2	–	2	2	–	4	–	5
10. *L*. *amansis* HI (MH730029)	42	42	42	45	43	41	46	2	1	–	–	–	–	–	–	–	–	–	–	–	–
11. *L*. sp. 5 HI (MH730050)	39	39	39	43	43	44	48	38	39	40	–	2	2	6	–	6	4	–	2	–	9
12. *L*. *amblyglyphidodonis* HI (MH730035)	44	44	44	46	48	45	58	43	43	43	36	–	0	6	–	6	4	–	2	–	9
13. *L*. *amblyglyphidodonis* HI (MH730033)	44	44	44	46	48	45	58	43	43	43	36	0	–	6	–	6	4	–	2	–	9
14. *L*. *cirripectis* LI (MH730041)	49	49	50	55	55	55	51	48	48	48	39	43	43	–	–	0	2	–	4	–	4
15. *L*. *cirripectis* LI (MH730040)	50	50	51	56	56	56	52	49	49	49	40	43	43	2	–	–	–	–	–	–	–
16. *L*. *cirripectis* HI (MH730036)	50	50	51	56	56	56	52	49	49	49	40	44	44	2	2	–	2	–	4	–	4
17. *L*. *adlardi* HI (MH730027)	44	44	44	47	47	46	54	40	40	40	41	48	48	48	49	47	–	–	4	–	5
18. *L*. *adlardi* HI (MH730028)	45	45	45	48	48	47	56	40	40	40	42	49	49	49	50	48	2	–	–	–	–
19. *L*. *acanthochromidis* HI (MH730025)	69	69	70	71	71	73	84	73	74	74	67	70	70	69	71	69	72	73	–	–	7
20. *L*. *melichthydis* Palau (MH730046)	64	64	64	64	64	63	67	60	60	60	60	62	62	66	67	67	56	56	70	–	–
21. *L*. *melichthydis* Palau (MH730047)	63	63	63	63	63	63	67	59	59	59	60	62	62	66	67	67	57	57	69	1	–

*Abbreviations*: MB, Moreton Bay; FP, Austral Islands, French Polynesia; HI, Heron Island; LI, Lizard Island

All data, molecular, morphological and biological (principally host distribution), were considered iteratively. Overall, the distinctions suggested by ITS2 and *cox*1 sequences are consistent with those suggested by host distribution and morphology. The 11 main genotypes/clades are thus considered to represent 10 species. The disparity between 11 genotypes/clades and the recognition of 10 species relates to the samples from *Hemitaurichthys polylepis* from Palau and the Austral and Marquesas Archipelagos in French Polynesia. Of the three sources of evidence available, host clearly gave no basis for distinction between these forms. The ITS2 distinction (a single base) was unique; no other putative species showed any intraspecific variation in ITS2 sequence data. The *cox*1 distinction was at a level lower than between any combination of species but far greater than within any of the other species which exhibited intraspecific variation. However, no other species was sequenced over such a wide geographical range, so we are unable to interpret this distinction in context. There was no difference in the partial 28S sequence data for samples from Palau and the Austral Archipelago. The morphology of the forms from *H. polylepis* suggests subtle distinctions, but nothing that amounts to a reliable difference. In the face of these combined data, we propose a conservative approach, interpreting all specimens from *H. polylepis* as a single species that demonstrates geographical genetic variation.

**Species of** ***Lepotrema***

***Lepotrema clavatum*** **Ozaki, 1932**

*Type-host*: *Stephanolepis cirrhifer* (Temminck & Schlegel) (Tetraodontiformes: Monacanthidae), threadsail filefish.

*Type-locality*: “Otaru southwards to Nagasaki”, Japan.

*Material studied*: Voucher specimens collected by Ozaki, probably the type-series, 3 slides with 10 worms, three mature and measured. Worms flattened.

*Locality*: Off Hiroshima, Hiroshima Prefecture, Japan.

*Voucher specimens*: Meguro Parasitological Museum: Vouchers 30029, 30030.

*Site in host*: Upper part of intestine.

*Records*: 1. Ozaki (1932); 2. Yamaguti (1934); 3. Yamaguti (1938); 4. Hanson (1955); 5. Pritchard (1963); 6. Ichihara (1968); 7. Yamaguti (1970); 8. Dyer et al. (1988); 9. Bray et al. (1993); 10. Machida & Kuramochi (1999); 11. Machida & Uchida (2001); 12. Kondo et al. (2016).

*Definitive hosts*: Monacanthidae: *Stephanolepis cirrhifer* (Temminck & Schlegel) (1, 2, 3, 6), *Thamnaconus modestus* (Günther) (2, 3, 9, 10).

*Doubtful definitive hosts*: Balistidae: *Melichthys niger* (Bloch) (4, 5), *Melichthys vidua* (Richardson) (5, 7), *Odonus niger* (Rüppell) (10), *Rhinecanthus aculeatus* (Linnaeus) (8); Chaetodontidae: *Hemitaurichthys zoster* (Bennett) (5); Paralichthyidae: *Pseudorhombus cinnamoneus* (Temminck & Schlegel) (2); Pomacanthidae: *Genicanthus semifasciatus* (Kamohara) (11); Pomacentridae: *Dascyllus albisella* Gill (5).

*Second intermediate hosts*: Cnidaria, Scyphozoa: *Aurelia aurita* (Linnaeus) (*s.l*.) (12), *Chrysaora pacifica* (Goette) (12), *Cyanea nozakii* Kishinouye (12).

*Freshly ingested immatures*: Centrolophidae: *Psenopsis anomala* (Temminck & Schlegel) (12); Monacanthidae: *Thamnaconus modestus* (12).

*Localities*: Japan (1, 2, 3, 6, 8, 9, 10, 11, 12), Hawaii (4, 5, 7).

### Remarks

**Figs. 3–7:**
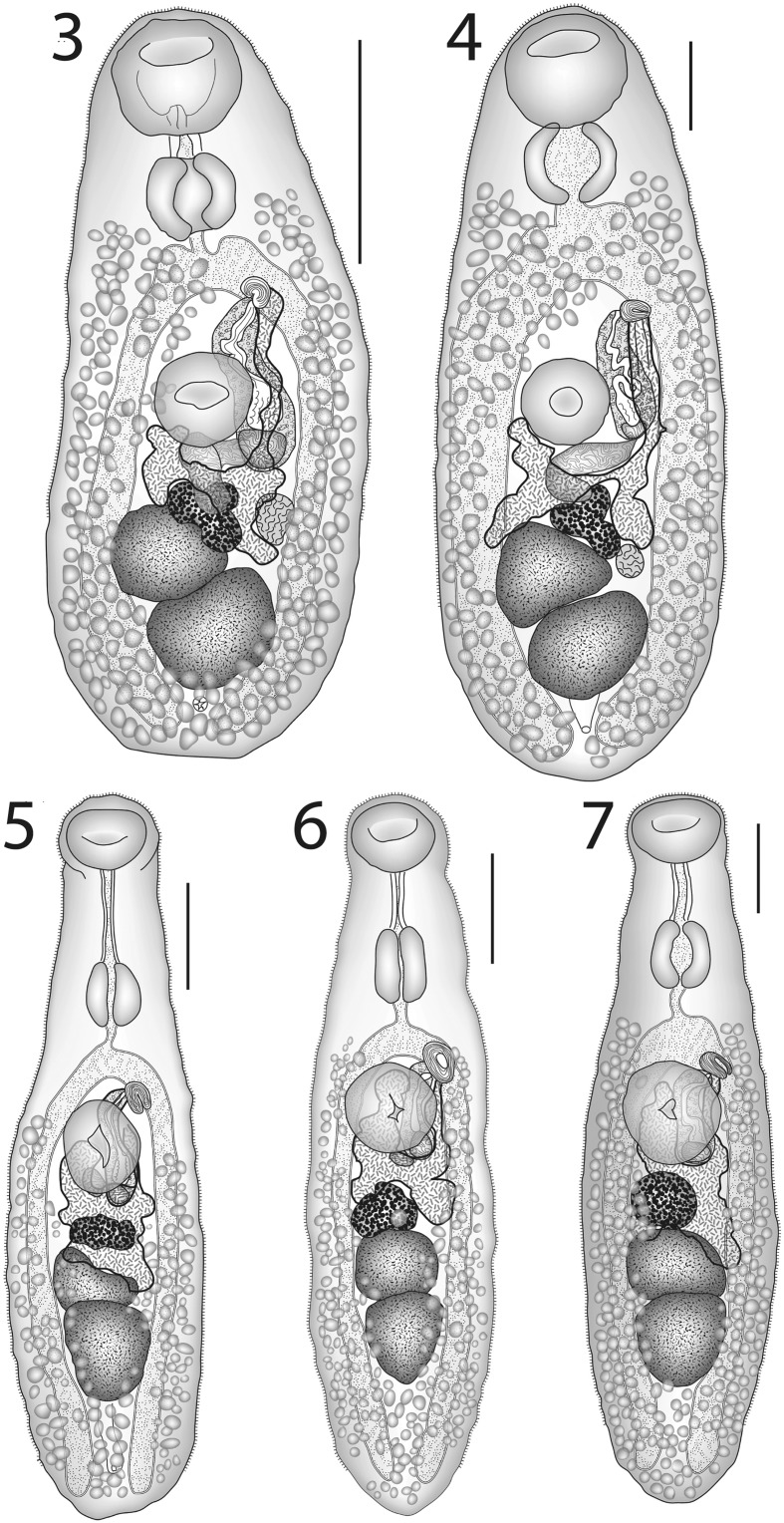
*Lepotrema* spp. 3, 4, *Lepotrema clavatum* Ozaki, 1932; 3, Ventral view of voucher in probable type-series; 4, Ventral view, redrawn from Ozaki (1932); 5–7, *Lepotrema adlardi* (Bray, Cribb & Barker, 1993); 5, ex *Abudefduf bengalensis*, Ningaloo Reef, ventral view; 6, ex *Abudefduf bengalensis*, off Heron Island, ventral view; 7, ex *Abudefduf bengalensis*, off Lizard Island, ventral view. *Scale-bars*: 200 μm

Hitherto, this, the type-species of *Lepotrema*, was known mainly from its original description. We reproduce here one new illustration (Fig. 3), a copy of the original illustration (Fig. 4) and give measurements for three specimens from the type-host, probably from the type-series. Molecular and morphometric results presented in this paper provide substantial doubt to the other records of this species (especially those from non-tetraodontiforms), given the overall pattern of oioxenous or stenoxenous host-specificity recognised here. Although we do not have molecular data for this species from its type-host and locality, despite the examination of nine specimens of the type-host from off Minabe, Wakayama Prefecture, Japan, we can be confident that some subsequent reports of this species were mistaken. The clearest evidence for this comes from the forms from *Acanthochromis polyacanthus*, *Amanses scopas* and *Melichthys vidua* from the Great Barrier Reef (Bray et al., 1993; Barker et al., 1994; Bray & Cribb, 1996c, 2002; Bray et al., 2009b), originally identified as *L. clavatum*, but which our molecular results indicate are separate species, differing by 98–100 bp in the partial *cox*1 dataset; clearly they could not relate to the true *L. clavatum* and it is our view that they do not represent *L. clavatum* and are distinct. These records, along with the record from *Parma polylepis* (see *Lepotrema* sp. 4 below), have been deleted from the list of hosts for this species. We base our ideas on the morphology of this worm on the original description (Ozaki, 1932) and our observations of ten worms, including three ovigerous worms from the type-host, probably the type series. A prepharynx was not described or illustrated by Ozaki (1932), but our observations indicate that a distinct prepharynx is present. The distinctive characters of this species include its large size, large oral sucker, and the relatively short pre-bifurcal and pre-vitelline distances (Table 2). In our view, it is highly probable that several more of the reports of *L. clavatum* will prove spurious, but more work is necessary to explore this.

**Table 2:** Dimensions of *Lepotrema clavatum* Ozaki, 1932, *sensu stricto* and *sensu lato*

Species	*Lepotrema clavatum*	*Lepotrema clavatum*	*Lepotrema* ‘*clavatum*’			
Host	*Monacanthus cirrhifer*	*Monacanthus cirrhifer*	*Monacanthus cirrhifer*; *Thamnaconus modestus*	*Pseudorhombus cinnamoneus*	*Genicanthus semifasciatus*	*Melichthys vidua*
Locality	Japan	Japan	Japan	Japan	Japan	Hawaii
Source	3 from “type-series”	Ozaki (1932)	Yamaguti (1934)	Yamaguti (1934)	Machida & Uchida (2001)	Yamaguti (1970)
			No illustration	No illustration	No illustration	No complete illustration
Body	1,525–2,083 × 590–872 (1,744 × 712)	1,690–1,785 × 650–750	1,870–1,920 × 670	1,430–1,580 × 470–550	1,450–2,150 × 670–950	870–1,360 × 350–540
Forebody	737–975 (824)	815^a^	–	–	630–1,111	–
Pre-oral lobe	16–19 (18)	16.5^a^	–	–	–	–
Oral sucker	229–270 × 262–342 (252 × 296)	250–300 × 250–300	240–280 × 280–330	240–260 × 260–340	120–170 × 160–250	120–160 × 160–220
Prepharynx	28–75 (52)	0^a^	–	–	100–150	–
Pharynx	131–221 × 193–281 (172 × 223)	160–180 × 160–180	170–190 × 210–220	150–190 × 190–210	100–150 × 110–230	90–150 × 150–210
Oesophagus	49–70 (61)	12^a^	–	–	50–120	60–100
Intestinal bifurcation to ventral sucker	216–364 (265)	354^a^	–	–	–	–
Pre-vitelline distance	434–520 (465)	384^a^	–	–	–	–
Vitellarium to ventral sucker	298–455 (359)	431^a^	–	–	–	–
Ventral sucker	199–252 × 198–253 (219 × 227)	170–200 × 170–200	260 × 260	240–280 × 230–260	200–290 × 200–290	120–180 × 120–180
Cirrus-sac	378–459 × 107–140 (420 × 125)	300 × 108^a^	–	–	450–600 × 120–140	100–340 × 40–70
Ventral sucker to ovary	55–129 (85)	92^a^	–	–	–	–
Ovary	132–197 × 137–184 (162 × 162)	162 × 162^a^	110–170 × 150–200	84–180 × 120–210	170–190 × 190–260	80–110 × 30–140
Ovary to anterior testis	0	0^a^	–	–	–	–
Anterior testis	205–254 × 215–344 (228 × 274)	246 × 265^a^	220–240 × 280–370	190–290 × 220–300	110–250 × 110–230	80–200 × 70–180
Distance between testes	0	0	–	–	–	–
Posterior testis	231–294 × 227–376 (257 × 294)	246 × 269^a^	220–240 × 280–370	190–290 × 220–300	110–250 × 110–230	80–200 × 70–180
Post-testicular distance	78–176 (141)	185^a^	–	–	–	–
Post-caecal distance	29–50 (30)	46^a^	–	–	–	–
Eggs	55–67 × 27–42 (59 × 34)	not given	51–58 × 34–37	57 × 32	48–58 × 30–33	39–55 × 29–30
Width (%)^b^	38.7–41.9 (40.7)	38.5–39.5	34.9–35.8^a^	32.9–34.8	44.2–46.2	39.7–40.2
Forebody (%)^b^	46.7–48.3 (47.3)	45.7^a^	–	–	44–47	–
Sucker length ratio	1:0.81–0.93 (0.87)	1:0.67–0.68	1:0.93–1.08	1:1.00–1.08	1:1.67–1.71	1:1.00–1.12
Sucker width ratio	1:0.74–0.81 (0.77)	1:0.67–0.68	1:0.79–0.93	1:0.76–0.88	1:1.12–1.37	1:0.75–0.82
Oral sucker: pharynx width	1:1.22–1.45 (1.34)	1:1.56–1.67	1:1.33–1.50	1:1.37–1.62	1:1.09–1.45	1:1.05–1.07
Ventral sucker to ovary (%)^b^	3.62–6.19 (4.71)	5.15^a^	–	–	–	–
Post-testicular distance (%)^b^	5.11–10.8 (8.00)	10.4^a^	–	–	–	–
Prepharynx (%)^b^	1.33–4.89 (3.18)	0^a^	–	–	6.90–7.00	–
Oesophagus (%)^b^	3.04–4.25 (3.55)	0.67^a^	–	–	3.45–5.58	
Intestinal bifurcation to ventral sucker distance (%)^b^	13.3–17.5 (15.0)	19.8^a^	–	–	–	–
Vitellarium to ventral sucker distance (%)^b^	19.5–21.8 (20.4)	24.1^a^	–	–	–	–
Ovary to anterior testis (%)^b^	0	0^a^	–	–	–	–
Distance between testes (%)^b^	0	0^a^	–	–	–	–
Cirrus-sac length (%)^b^	22.0–260 (24.3)	16.8^a^	–	–	27.9–31.0	11.5–25.0
Pre-vitelline distance (%)^b^	25.0–28.8 (26.8)	22.5^a^	–	–	–	–
Anterior testis length (%)^b^	12.2–13.9 (13.2)	13.8^a^	11.8–12.5	13.3–18.4	7.59–11.6	9.20–14.7
Posterior testis length (%)^b^	14.1–15.2 (14.8)	7.59–11.6	9.20–14.7	14.9–16.7	13.3–21.0	12.4^a^

^a^From the illustration; ^b^%, percent of body length

***Lepotrema adlardi*** **(Bray, Cribb & Barker, 1993) Bray & Cribb, 1996**

Syn. *Lepocreadium adlardi* Bray, Cribb & Barker, 1993

*Type-host*: *Abudefduf bengalensis* (Bloch) (Perciformes: Pomacentridae), Bengal sergeant.

*Type-locality*: Off Heron Island, Great Barrier Reef, Australia.

*Records*: 1. Bray et al. (1993); 2. Barker et al. (1994); 3. Present study.

*Host*: Pomacentridae: *Abudefduf bengalensis* (1, 2, 3).

*New material*

*Host*: *Abudefduf bengalensis*.

*Localities*: Off Heron Island (23°27′S, 151°55′E), off Lizard Island (14°40′S, 145°28′E), Queensland, Australia; Ningaloo Reef (22°42′S, 113°40′E), Western Australia.

*Prevalence*: Off Heron Island: in 20 of 43 fish examined; off Lizard Island: in 2 of 5 fish examined; Ningaloo Reef: in 3 of 13 fish examined.

*Voucher material*: Off Heron Island (QM G237457–9; NHMUK 2018.7.23.1); off Lizard Island (QM G237460); Ningaloo Reef (QM G237461–3; WAM V9310-3; NMHUK 2018.7.23.2)

*Representative DNA sequences*: ITS2 rDNA, two identical replicates (one submitted to GenBank MH730000); *cox*1 mtDNA, two replicates (both submitted to GenBank MH730027–28); 28S rDNA, one sequence (submitted to GenBank MH730015).

### Remarks

This species is morphologically and genetically distinct, being narrow, with an even narrower, long forebody, a long prepharynx and a pre-vitelline distance similar to the forebody length (Figs. 5–7). New measurements are given in Table 3. This is the first report of *L. adlardi* from the northern Great Barrier Reef (Lizard Island) and the Indian Ocean (Ningaloo Reef). The small samples sizes available (Table 3) give no evidence of morphological variation between the localities. The sequenced specimens come only from the type-locality. The species appears strictly oioxenic to *Abudefduf bengalensis*. Thirty-two specimens of *A. bengalensis* have been examined in Moreton Bay, but this species has never been recovered from there. It has also never been found in the banded sergeant *A. septemfasciatus* (Cuvier) (8 specimens examined, from Lizard Island and French Polynesia), the scissortail sergeant *Abudefduf sexfasciatus* (Lacépède) (65 specimens from many localities), the blackspot sergeant *A. sordidus* (Forsskål) (6 specimens from various localities), the Indo-Pacific sergeant *A. vaigiensis* (Quoy & Gaimard) (12 specimens from the GBR and Moreton Bay) or Whitley’s sergeant *A. whitleyi* Allen & Robertson (308 specimens from various localities). Forty-one other pomacentrid species have been investigated without the recovery of *L. adlardi*.

**Table 3:** Dimensions of *Lepotrema adlardi* (Bray, Cribb & Barker, 1993) and *L. acanthochromidis* n. sp.

Species	*Lepotrema adlardi*			*Lepotrema acanthochromidis*	
Host	*Abudefduf bengalensis*			*Acanthochromis polyacanthus*	
Locality	Ningaloo Reef	Heron Island	Lizard Island	Heron Island	Lizard Island
n	5	5	1	9	17
Body	1,293–1,683 × 373–536 (1,486 × 441)	1,103–1491 × 252–359 (1,269 × 301)	1,636 × 448	830–1,680 × 340–674 (1,160 × 442)	914–1,510 × 338–743 (1,194 × 505)
Forebody	477–653 (570)	475–608 (513)	614	343–571 (439)	364–556 (452)
Pre-oral lobe	21–23 (22)	9–24 (16)	12	3–28 (14)	7–28 (15)
Oral sucker	114 –133 × 151–165 (122 × 159)	101–148 × 124–154 (120 × 141)	142 × 201	94–158 × 140–223 (121 × 165)	95–172 × 150–206 (131 × 178)
Prepharynx	94–189 (148)	98–178 (128)	128	25–57 (37)	0–66 (38)
Pharynx	122–152 × 109–118 (136 × 114)	96–143 × 70–106 (122 × 91)	158 × 134	74–130 × 67–128 (91 × 97)	71–128 × 79–140 (96 × 105)
Oesophagus	33–53 (42)	49–75 (61)	75	35–74 (48)	23–49 (37)
Intestinal bifurcation to ventral sucker	86–128 (107)	57–88 (74)	99	86–160 (127)	88–200 (136)
Pre-vitelline distance	423–588 (523)	412–561 (466)	521	227–312 (256)	199–307 (259)
Vitellarium to ventral sucker	0–95 (47)	33–63 (47)	93	111–259 (183)	143–269 (192)
Ventral sucker	178–219 × 156–197 (195 × 172)	124–174 × 124–175 (151 × 149)	235 × 226	105–194 × 113–202 (133 × 143)	119–177 × 113–190 (146 × 154)
Cirrus-sac	191–361 × 62–90 (266 × 79)	152–237 × 56–76 (188 × 64)	275 × 82	209–277 × 61–94 (242 × 74)	211–302 × 52–92 (253 × 76)
Ventral sucker to ovary	22–92 (53)	33–84 (54)	14	0–97 (52)	0–59 (32)
Ovary	88–189 × 80–161 (128 × 118)	82–110 × 62–144 (97 × 83)	129 × 154	76–172 × 100–174 (116 × 132)	78–193 × 94–197 (109 × 131)
Ovary to anterior testis	0–2 (0)	0	0	0	0
Anterior testis	119–172 × 115–182 (135 × 156)	95–123 × 104–159 (111 × 123)	170 × 213	93–203 × 99–218 (121 × 141)	101–223 × 109–298 (151 × 173)
Distance between testes	0	0	0	0	0
Posterior testis	148–223 × 118–164 (185 × 148)	108–164 × 102–155 (137 × 121)	215 × 203	104–221 × 101–186 (144 × 138)	112–257 × 112–244 (178 × 160)
Post-testicular distance	229–306 (267)	164–304 (225)	267	130–248 (190)	119–275 (184)
Post-caecal distance	36–67 (52)	39–64 (55)	75	65–101 (79)	28–96 (65)
Eggs	53–61 × 26–37 (56 × 31)	51–60 × 32–41 (55 × 37)	59 × 26	58–71 × 32–41 (65 × 36)	49–68 × 27–36 (55 × 32)
Width (%)^a^	27.3–32.0 (29.6)	22.5–25.4 (23.7)	27.4	34.5–42.0 (38.1)	34.7–51.9 (42.1)
Forebody (%)^a^	36.6–420 (38.3)	37.0–43.0 (40.6)	37.6	34.0–41.3 (38.3)	35.3–42.7 (38.0)
Sucker length ratio	1:1.40–1.92 (1.60)	1:0.94–1.43 (1.28)	1:1.66	1:0.95–1.23 (1.10)	1:0.93–1.32 (1.13)
Sucker width ratio	1:0.94–1.22 (1.08)	1:0.93–1.15 (1.06)	1:1.12	1:0.81–0.91 (0.86)	1:0.76–0.98 (0.86)
Oral sucker: pharynx width	1:1.29–1.49 (1.40)	1:1.38–1.91 (1.59)	1:1.50	1:1.52–2.09 (1.72)	1:1.42–2.06 (1.73)
Ventral sucker to ovary (%)^a^	1.71–5.44 (3.40)	2.97–5.66 (4.18)	0.86	0–7.77 (4.20)	0–5.11 (2.71)
Post-testicular distance (%)^a^	16.4–20.0 (18.1)	14.9–20.4 (17.6)	16.3	14.8–18.1 (16.4)	10.9–21.7 (15.4)
Prepharynx (%)^a^	7.65–12.6 (10.5)	7.29–12.6 (10.1)	7.83	1.83–5.80 (3.28)	0–7.01 (3.28)
Oesophagus (%)^a^	2.37–3.17 (2.81)	3.69–6.27 (4.84)	4.56	3.16–5.48 (4.13)	1.97–4.84 (3.11)
Intestinal bifurcation to ventral sucker distance (%)^a^	6.67–7.89 (7.19)	4.75–7.28 (5.84)	6.04	9.36–12.9 (11.1)	7.63–14.0 (11.4)
Vitellarium to ventral sucker distance (%)^a^	0–5.62 (2.98)	2.70–5.71 (3.81)	5.69	13.3–17.3 (15.8)	12.8–18.8 (16.1)
Ovary to anterior testis (%)^a^	0–0.12 (0.02)	0	0	0	0
Distance between testes (%)^a^	0	0	0	0	0
Cirrus-sac length (%)^a^	13.8–21.6 (17.6)	13.7–15.9 (14.8)	16.8	16.5–27.0 (21.3)	18.6–29.8 (21.5)
Pre-vitelline distance (%)^a^	32.3–42.0 (35.4)	34.3–38.1 (36.7)	31.9	18.6–28.0 (22.5)	18.5–25.4 (22.0)
Anterior testis length (%)^a^	7.54–10.3 (9.07)	7.74–9.78 (8.80)	10.4	9.39–12.1 (10.4)	9.73–14.9 (12.6)
Posterior testis length (%)^a^	10.7–13.3 (12.4)	8.97–12.2 (10.8)	13.1	9.94–14.2 (12.4)	12.2–17.2 (14.7)

^a^%, percent of body length

***Lepotrema acanthochromidis*** **n. sp.**

Syn. *L. clavatum* of Bray et al. (1993), Barker et al. (1994) in part

*Type-host*: *Acanthochromis polyacanthus* (Bleeker) (Perciformes: Pomacentridae), spiny chromis.

*Type-locality*: Off Heron Island (23°27′S, 151°55′E), Great Barrier Reef, Australia.

*Other locality*: Off Lizard Island (14°40′S, 145° 28′E), Great Barrier Reef, Australia.

*Type-material*: Off Heron Island: holotype (QM GL 14769); paratypes (QM GL 14770–72, G237464–70; NHMUK 2018.7.23.3–4); off Lizard Island (QM G237471–82; NHMUK 2018.7.23.5–9).

*Site in host*: Intestine.

*Prevalence*: Off Heron Island: in 21 of 65 fish examined; off Lizard Island: in 21 of 74 fish examined.

*Representative DNA sequences*: ITS2 rDNA, two identical replicates (one submitted to GenBank MH729999); *cox*1 mtDNA, two identical replicates (both submitted to GenBank MH730025–26); 28S rDNA, one sequence (submitted to GenBank MH730014).

*ZooBank registration*: To comply with the regulations set out in article 8.5 of the amended 2012 version of the *International Code of Zoological Nomenclature* (ICZN, 2012), details of the new species have been submitted to ZooBank. The Life Science Identifier (LSID) for *Lepotrema acanthochromidis* n. sp. is urn:lsid:zoobank.org:act:923FD389-26CA-412A-980A-6C6B7116BF63.

*Etymology*: The specific epithet is derived from the generic name of the host species.

*Previous records*: Pomacentridae: *Acanthochromis polyacanthus*, off Heron Island (Bray et al., 1993, as *L. clavatum*; Barker et al., 1994); off Lizard Island (Bray et al., 2009b, as *L. clavatum*).

### Description (Figs. 8–9)

[Based on 26 whole-mounted specimens, 9 from off Heron Island, 17 from off Lizard Island; measurements in Table 3.] Body elongate-oval. Tegument finely spined; spines reaching to about ovarian level. Oral sucker transversely oval, subterminal. Ventral sucker oval, of similar length to, but distinctly narrower than oral sucker, pre-equatorial. Prepharynx usually distinct, short, thick-walled. Pharynx oval. Oesophagus short, narrow. Intestinal bifurcation in posterior forebody. Caeca broad, reach into post-testicular region.

**Figs. 8–9:**
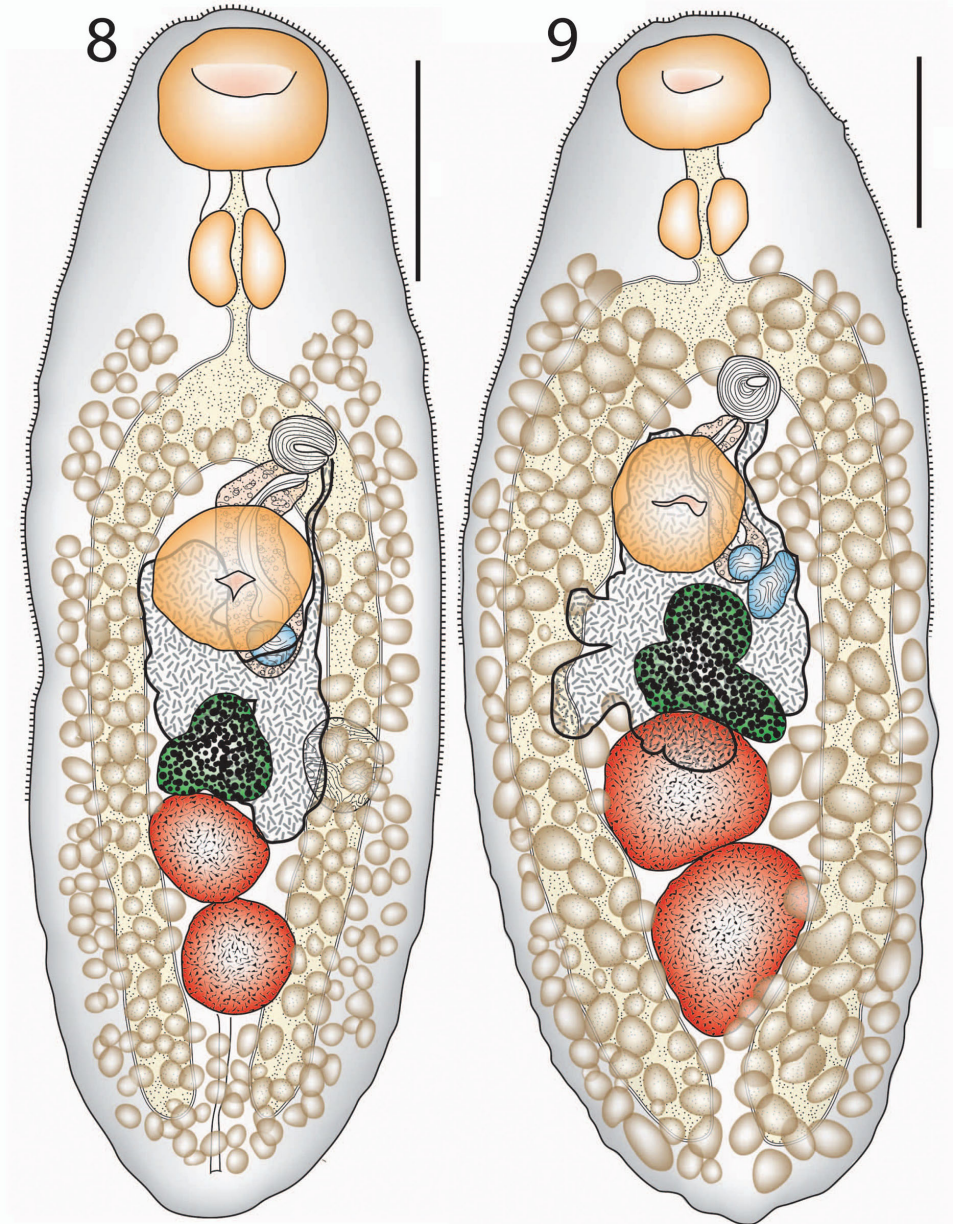
*Lepotrema acanthochromidis* n. sp. 8, ex *Acanthochromis polyacanthus*, off Heron Island, holotype, ventral view; 9, ex *Acanthochromis polyacanthus*, off Lizard Island, ventral view. *Scale-bars*: 200 μm

Testes 2, oval, entire, virtually tandem or slightly oblique, in mid-hindbody. External seminal vesicle usually obscured by eggs, but small when seen. Cirrus-sac claviform, mainly dorsal to ventral sucker. Internal seminal vesicle rounded to oval. Pars prostatica vesicular. Ejaculatory duct long, muscular. Genital atrium distinct. Genital pore sinistral, ventral to sinistral caecum at bifurcal level or just posterior.

Ovary trilobate, immediately pre-testicular, close or adjacent to ventral sucker. Laurer’s canal opens at about level of anterior edge of anterior testis. Seminal receptacle dorsal or dorso-lateral to ovary. Mehlis’ gland dorsal to ovary or anterior part of anterior testis. Uterus intercaecal, mostly pre-testicular, passes ventrally to ovary, overlaps ventral sucker. Eggs tanned, operculate. Metraterm shorter than cirrus-sac, distal extremity with large folded muscular pad. Vitellarium follicular, reaching from posterior edge of pharynx to posterior extremity, fields may be confluent in forebody (as narrow band) and post-testicular region; lateral and ventral to caeca.

Excretory pore dorsal, in anterior post-testicular region; vesicle reaches to testes, not traced further.

### Remarks

This species is characterised by molecular means (Table 1) and distinguished from similar congeners by the following morphological characteristics (Table 3). *Lepotrema clavatum* is larger, with a relatively longer forebody, relatively larger oral sucker and pharynx, a longer cirrus-sac, a shorter post-testicular region, longer ventral sucker to bifurcal distance and ventral sucker to ovary distances, a slightly shorter pre-vitelline distance, a smaller sucker ratio and slightly longer caeca. *Lepotrema incisum* has deeply incised testes, a relatively larger pharynx, a longer pre-vitelline distance and cirrus-sac, a relatively shorter post-testicular region and smaller eggs. *Lepotrema monile* lacks a strong muscular pad around the metraterm, has a small sphincter and has a relatively shorter cirrus-sac.

The two worms sequenced from off Heron Island have identical ITS2 and *cox*1 sequence data. 28S sequence data for the specimens from off Heron Island differed from samples from off Lizard Island (GenBank: FJ788483.1) by 2 bp; this is a level greater than between conspecific *Lepotrema* samples infecting *Hemitaurichthys polylepis* from Palau and French Polynesia, which had identical 28S data across these regions. In addition, specimens of *L. acanthochromidis* n. sp. from off Heron Island tend to have slightly longer eggs than those from off Lizard Island. However, given the host and generally similar morphology and the lack of ITS2 and *cox*1 data for samples from off Lizard Island, we take a conservative approach and recognise both sets of samples as the same species. This anomaly is worthy of further study.

This species has strongly oioxenous specificity for *Acanthochromis polyacanthus*, having been found in that species at least 40 times on the GBR but never in 1,228 individuals of 55 other pomacentrid species examined on the GBR. *Acanthochromis polyacanthus* can be found together with *Abudefduf bengalensis* (the host of *L. adlardi*) and *Pomacentrus wardi* (the host of *L. monile*, see below) but there is no evidence of any sharing of the three *Lepotrema* species by these three pomacentrid species. Notably, according to Cribb et al. (1994), *A. polyacanthus* is also the only (but frequently infected) pomacentrid host for a bivesiculid, *Bivesicula unexpecta* Cribb, Bray & Barker, 1994.

***Lepotrema hemitaurichthydis*** **n. sp.**

*Type-host*: *Hemitaurichthys polylepis* (Bleeker) (Perciformes: Chaetodontidae), pyramid butterflyfish.

*Other host*: *Hemitaurichthys thompsoni* Fowler (Perciformes: Chaetodontidae), Thompson’s butterflyfish.

*Type-locality*: Off Palau (07°30′N, 134°30′E).

*Other localities*: Ex *H. polylepis*: off Tubuai (23°22′S, 149°28′W), off Rimatara (22°39′S, 152°49′W), Austral Islands, French Polynesia; ex *H. thompsoni*: off Fatu Hiva, Marquesas, French Polynesia (10°27′S, 138°40′W).

*Type-material*: Holotype (QM G237483), paratypes: ex *H. polylepis* off Palau (QM G237484–91; NMHUK 2018.7.23.10–13); off Tubuai (QM G237492–3; NHMUK 2018.7.23.14); off Rimatara (QM G237494). Voucher: ex *H. thompsoni* (QM G237495).

*Site in host*: Intestine.

*Prevalence*: Ex *H. polylepis*: off Palau (in 13 of 15 fish examined); off Tubuai (in 2 of 5 fish examined); off Rimatara (in 1 fish examined). Ex *H. thompsoni*: off Fatu Hiva (in 2 of 3 fish examined).

*Representative DNA sequences*: ITS2 rDNA, four replicates (two submitted to GenBank MH730006–07); *cox*1 mtDNA, four replicates (all submitted to GenBank MH730042–45); 28S rDNA, two identical replicates (both submitted to GenBank MH730019–20).

*ZooBank registration*: To comply with the regulations set out in article 8.5 of the amended 2012 version of the *International Code of Zoological Nomenclature* (ICZN, 2012), details of the new species have been submitted to ZooBank. The Life Science Identifier (LSID) for *Lepotrema hemitaurichthydis* n. sp. is urn:lsid:zoobank.org:act:FD097AD4-0E9E-4648-9AE7-673AC9701552.

*Etymology*: The specific epithet is derived from the generic name of the host species.

### Description (Figs. 10–14)

[Based 18 whole-mounted specimens, 17 ex *H. polylepis*, 13 from off Palau, 3 from off Tubuai and 1 from off Rimatara, and 1 ex *H. thompsoni*; measurements in Table 4.] Body oval or slightly pyriform, slightly wider in hindbody. Tegument finely spined, spines reaching to, or close to, posterior extremity. Oral sucker large, transversely oval, subterminal. Ventral sucker rounded to oval, usually smaller than oral sucker, pre-equatorial. Prepharynx absent to short. Pharynx large, oval to subglobular. Oesophagus short, narrow. Intestinal bifurcation in posterior forebody. Caeca broad, reach to about middle of post-testicular region or beyond.

**Figs. 10–14:**
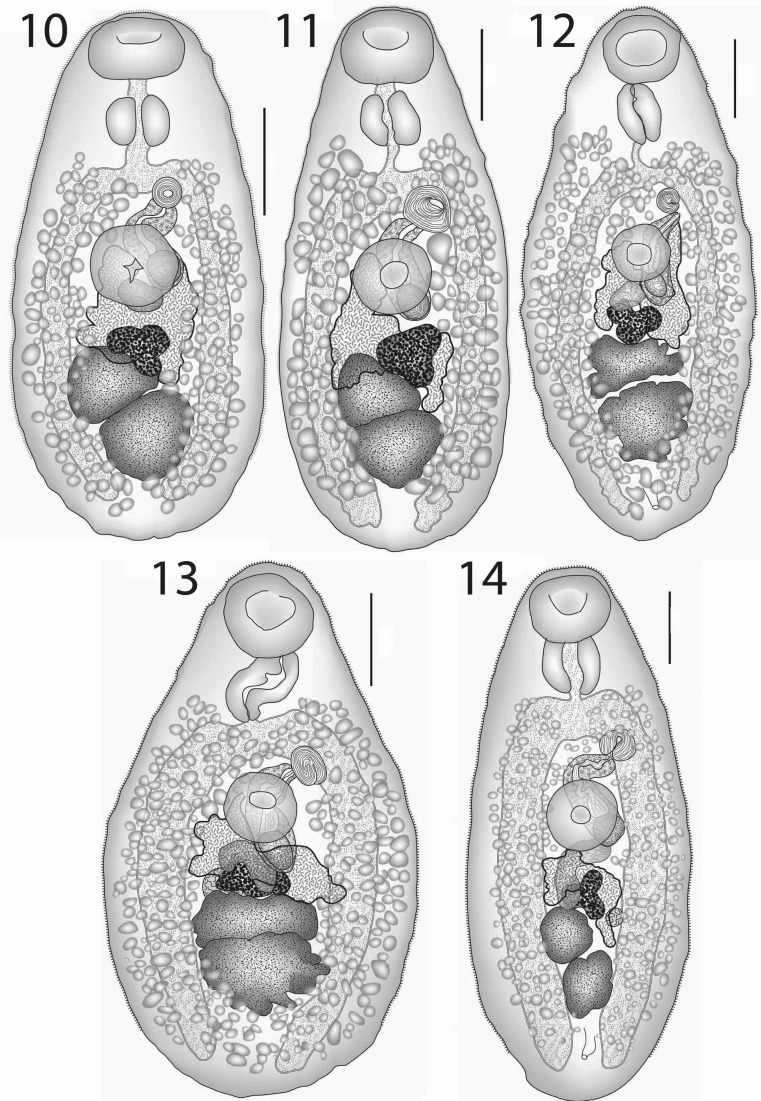
*Lepotrema hemitaurichthydis* n. sp. 10, ex *Hemitaurichthys polylepis*, Palau, holotype, ventral view; 11, ex *Hemitaurichthys polylepis*, off Tubuai, Austral Islands, ventral view; 12, ex *Hemitaurichthys polylepis*, off Tubuai, Austral Islands, paratype with lobate testes; 13, ex *Hemitaurichthys polylepis*, off Tubuai, Austral Islands, paratype with lobate testes; 14, ex *Hemitaurichthys thompsoni*, Fatu Hiva, Marquesas, ventral view. *Scale-bars*: 200 μm

**Table 4:** Dimensions of *Lepotrema hemitaurichthydis* n. sp.

Host	*Hemitaurichthys polylepis*		*Hemitaurichthys thompsoni*
Locality	Australs, French Polynesia	Palau	Fatu Hiva, Marquesas
n	4	13	1
Body	1,145–1,355 × 506–677 (1,250 × 594)	798–1,140 × 381–655 (992 × 472)	1,470 × 588
Forebody	442–548 (507)	354–472 (410)	577
Pre-oral lobe	3–21 (13)	0–20 (8)	10
Oral sucker	159–180 × 195–212 (172 × 201)	103–142 × 155–210 (117 × 176)	189 × 223
Prepharynx	0–13 (6)	0–38 (22)	0
Pharynx	121–211 × 105–127 (167 × 117)	91–133 × 98–157 (105 × 125)	163 × 152
Oesophagus	18–64 (47)	20–50 (34)	30
Intestinal bifurcation to ventral sucker	99–181 (138)	86–203 (126)	214
Pre-vitelline distance	259–318 (290)	200–306 (251)	322
Vitellarium to ventral sucker	165–241 (217)	103–207 (159)	255
Ventral sucker	152–169 × 157–184 (162 × 173)	121–168 × 132–177 (143 × 155)	194 × 194
Cirrus-sac length	289–295 × 77–118 (291 × 96)	196–289 × 51–90 (239 × 73)	354 × 110
Ventral sucker to ovary	0–48 (22)	0–71 (26)	49
Ovary	97–129 × 137–162 (116 × 146)	59–104 × 64–137 (83 × 96)	136 × 122
Ovary to anterior testis	0	0–8 (1)	0
Anterior testis	120–163 × 195–264 (148 × 233)	94–163 × 94–192 (122 × 135)	161 × 150
Distance between testes	0	0	0
Posterior testis	164–213 × 200–264 (185 × 231)	115–221 × 93–185 (151 × 131)	189 × 144
Post-testicular distance	133–195 (166)	87–175 (124)	218
Post-caecal distance	26–62 (47)	33–64 (43)	70
Eggs	48–56 × 26–30 (50 × 28)	42–57 × 18–37 (51 × 31)	50 × 23
Width (%)^a^	43.3–59.1 (47.8)	42.5–58.1 (47.4)	40.0
Forebody (%)^a^	38.6–42.2 (40.6)	39.1–44.5 (41.4)	39.2
Sucker length ratio	1:0.87–1.06 (0.95)	1:1.09–1.35 (1.22)	1:1.03
Sucker width ratio	1:0.79–0.94 (0.87)	1:0.80–0.99 (0.88)	1:0.87
Oral sucker: pharynx width	1:1.53–1.90 (1.72)	1:1.25–1.58 (1.43)	1:1.46
Ventral sucker to ovary (%)^a^	0–3.51 (1.71)	0–6.66 (2.55)	3.35
Post-testicular distance (%)^a^	11.4–14.8 (13.3)	9.92–15.3 (12.4)	14.9
Prepharynx (%)^a^	0–1.08 (0.45)	0–3.80 (2.29)	0
Oesophagus (%)^a^	1.60–5.51 (3.74)	1.85–5.33 (3.52)	2.02
Intestinal bifurcation to ventral sucker distance (%)^a^	8.61–13.6 (11.0)	9.26–18.4 (12.6)	14.5
Vitellarium to ventral sucker distance (%)^a^	14.4–20.0 (17.4)	12.4–21.6 (16.0)	17.3
Ovary to anterior testis (%)^a^	0	0–0.79 (0.06)	0
Distance between testes (%)^a^	0	0	0
Cirrus-sac length (%)^a^	21.7–25.4 (23.4)	19.6–28.2 (24.1)	24.1
Pre-vitelline distance (%)^a^	22.2–24.1 (23.2)	21.9–31.6 (25.4)	21.9
Anterior testis length (%)^a^	10.5–12.6 (11.8)	10.7–15.3 (12.2)	11.0
Posterior testis length (%)^a^	14.1–15.7 (14.8)	12.4–21.6 (15.2)	12.8

^a^%, percent of body length

Testes 2, subtriangular entire or slightly irregular to distinctly lobed, virtually tandem to oblique, in mid hindbody. External seminal vesicle oval to elongate-saccular, often obscured by eggs. Cirrus-sac claviform, mainly dorsal to ventral sucker. Internal seminal oval. Pars prostatica vesicular. Ejaculatory duct long, muscular. Genital atrium distinct. Genital pore sinistral, ventral to sinistral caecum at bifurcal level or just post-bifurcal.

Ovary trilobate, immediately pre-testicular, adjacent or close to ventral sucker. Laurer’s canal opening dorsally at, or close to, sinistral edge of anterior testis. Seminal receptacle dorsal or dorso-lateral to ovary or overlapping anterior testis. Mehlis’ gland dorsal to ovary. Uterus intercaecal, mainly pre-testicular, passes ventrally to ovary, overlaps ventral sucker. Eggs tanned, operculate. Metraterm shorter than cirrus-sac, distal extremity with large folded muscular pad. Vitellarium follicular, reaching from pharynx to just into post-testicular region or close to posterior extremity, fields confluent or nearly so in forebody and post-testicular region; lateral and ventral to caeca.

Excretory pore dorsal, in anterior post-testicular region; vesicle reaches ovary.

### Remarks

The specimen from *H. thompsoni* bears a close resemblance to some of those from *H. polylepis* but is larger than any measured specimens from that species. The ratios of body-parts are similar to those in *H. polylepis* specimens and there seems no other reason why this form should be considered distinct. No sequence data are available for the *H. thompsoni* specimen. *Lepotrema hemitaurichthydis* n. sp. is characterised by molecular means (Table 1) and distinguished from similar congeners by the following morphological characteristics (Table 4). *Lepotrema clavatum* is usually larger, with a longer forebody, a distinct prepharynx, mostly smaller, shorter forebody, absent or short prepharynx (going by the voucher specimens we have examined, not the original description of *L. clavatum*), a smaller pharynx, a longer intestinal bifurcation to ventral sucker distance, a larger cirrus-sac, a longer ventral sucker to ovary distance, relatively shorter post-testicular and post-caecal distances and possibly slightly larger eggs. *Lepotrema acanthochromidis* n. sp. is relatively narrower, with a smaller pharynx and possibly ventral sucker, tending to have a longer prepharynx and slightly longer post-testicular and post-caecal distances. *Lepotrema incisum* has deeply incised testes, relatively larger pharynx and pre-vitelline distance, a shorter cirrus-sac, a relatively shorter post-testicular region and smaller eggs. *Lepotrema monile* has a small sphincter rather than a strong muscular pad around the metraterm and a relatively shorter cirrus-sac.

In our investigations of Indo-Pacific fishes, we have examined over 1,600 individuals of 35 species of chaetodontids. Species of *Lepotrema* have been found only in the two species *Hemitaurichthys* that we have examined. Multiple infections were detected in *Hemitaurichthys polylepis* off Palau (13 of 15) and in the Austral Archipelago of French Polynesia (3 of 6).

***Lepotrema melichthydis*** **n. sp.**

Syn. *Lepotrema clavatum* of Bray & Cribb (2002)

*Type-host*: *Melichthys vidua* (Richardson) (Tetraodontiformes: Balistidae), pinktail triggerfish.

*Type-locality*: Off Palau (07°30′N, 134°30′E).

*Other locality*: Off Heron Island (23°27′S, 151°55′E), Great Barrier Reef, Australia.

*Type-material*: Off Palau: holotype (QM G237496); paratypes (QM G237497–9; NHMUK 2018.7.23.15–16); off Heron Island (QM G237500–2; NHMUK 2018.7.23.17).

*Site in host*: Intestine.

*Prevalence*: Off Palau: in 1 fish examined; off Heron Island: in 1 fish examined.

*Representative DNA sequences*: ITS2 rDNA, two identical replicates (one submitted to GenBank MH730008); *cox*1 mtDNA, two replicates (both submitted to GenBank MH730046–47); 28S rDNA, one sequence (submitted to GenBank MH730021).

*ZooBank registration*: To comply with the regulations set out in article 8.5 of the amended 2012 version of the *International Code of Zoological Nomenclature* (ICZN, 2012), details of the new species have been submitted to ZooBank. The Life Science Identifier (LSID) for *Lepotrema melichthydis* n. sp. is urn:lsid:zoobank.org:act:A9D76B3A-5027-4D1E-8DE9-07393C124A5A.

*Etymology*: The specific epithet is derived from the generic name of the host species.

### Description (Figs. 15–16)

[Based on 12 whole-mounted specimens, 6 each from off Palau and Heron Island; measurements in Table 5.] Body oval. Tegument finely spined in forebody and anterior hindbody. Oral sucker oval to subglobular, subterminal. Ventral sucker rounded, of similar length but smaller width to oral sucker, pre-equatorial. Prepharynx absent to short, thick-walled. Pharynx large, longer than oral sucker, oval. Oesophagus short, narrow. Intestinal bifurcation in posterior forebody. Caeca broad, reach well into post-testicular region.

**Figs. 15–21:**
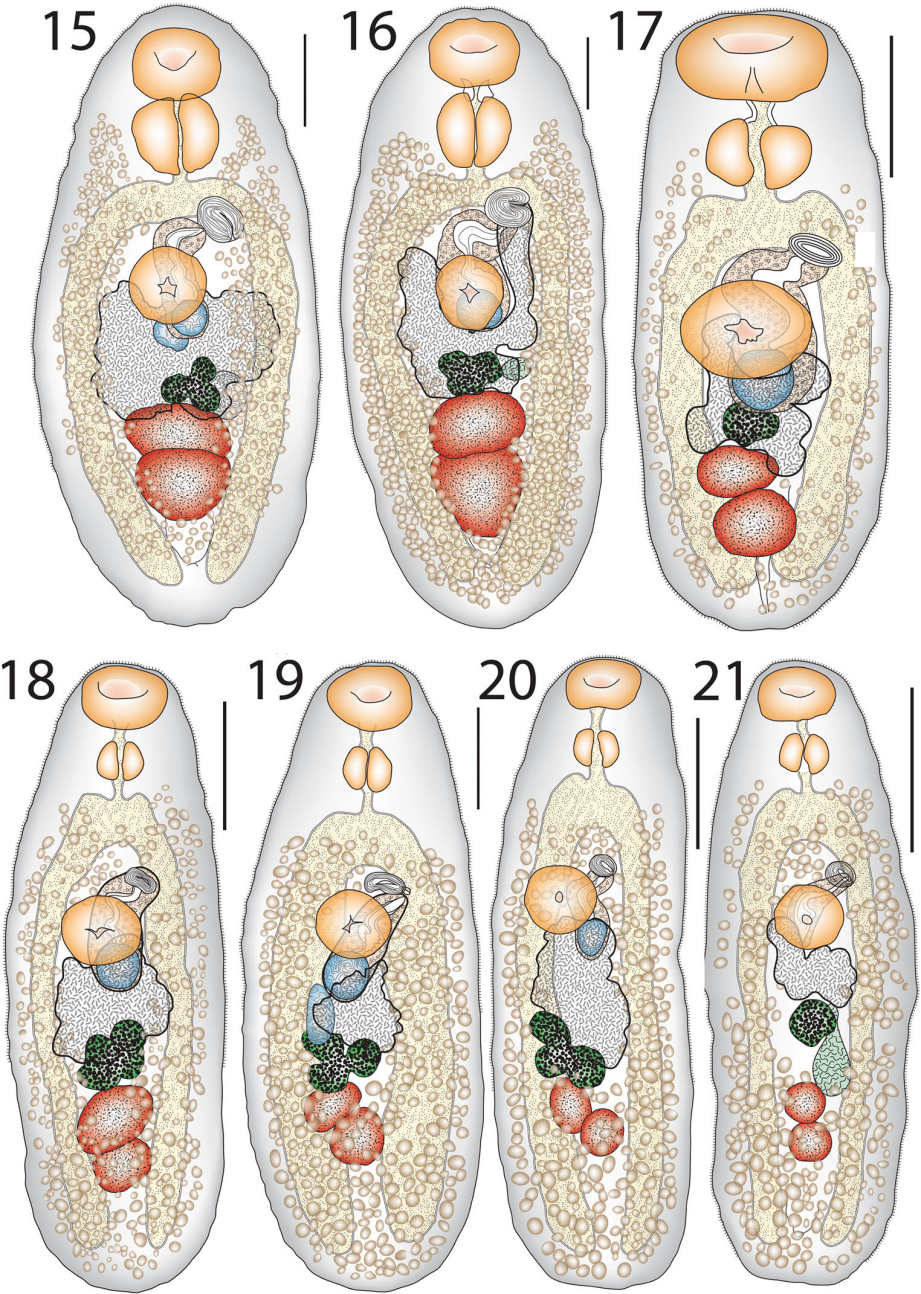
*Lepotrema* spp. 15, 16, *Lepotrema melichthydis* n. sp.; 15, ex *Melichthys vidua*, Palau, holotype, ventral view; 16, ex *Melichthys vidua*, off Heron Island, ventral view; 17, *Lepotrema amansis* n. sp. ex *Amanses scopas*, off Heron Island, holotype, ventral view; 18–21, *Lepotrema cirripectis* n. sp.; 18, 19, ex *Cirripectes filamentosus*, off Lizard Island; 18, Holotype, ventral view; 19, Paratype, ventral view; 20, ex *Cirripectes stigmaticus*, off Lizard Island, ventral view; 21, ex *Cirripectes chelomatus*, off Heron Island, ventral view. *Scale-bars*: 200 μm

**Table 5:** Dimensions of *Lepotrema melichthydis* n. sp., *L. amansis* n. sp. and *L. cirripectis* n. sp.

Species	*Lepotrema melichthydis*		*Lepotrema amansis*	*Lepotrema cirripectis*		
Host	*Melichthys vidua*		*Amanses scopas*	*Cirripectes filamentosus*	*Cirripectes stigmaticus*	*Cirripectes chelomatus*
Locality	Palau	Heron Island	Heron Island	Lizard Island		Heron Island
n	6	6	26	31	1	8
Body	1,042–1,504 × 424–660 (1,234 × 533)	1,183–1,928 × 480–889 (1,597 × 675)	621–955 × 221–403 (766 × 288)	765–1,507 × 275–608 (1,160 × 424)	989 × 302	562–704 × 178–243 (642 × 218)
Forebody	430–605 (490)	474–742 (625)	260–385 (316)	276–564 (411)	329	205–256 (236)
Pre-oral lobe	35–58 (45)	28–47 (37)	0–13 (5)	0–9 (2)	0	0–5 (1)
Oral sucker	107–162 × 150–216 (135 × 175)	149–198 × 187–272 (173 × 236)	70–139 × 118–178 (104 × 150)	76–156 × 111–209 (107 × 160)	79 × 128	52–67 × 74–91 (59 × 84)
Prepharynx	0–24 (9)	0–28 (14)	0–37 (22)	13–74 (31)	37	10–22 (14)
Pharynx	121–177 × 119–196 (143 × 149)	150–220 × 153–206 (193 × 182)	64–111 × 74–118 (87 × 93)	51–99 × 64–118 (76 × 91)	56 × 71	38–47 × 37–58 (43 × 51)
Oesophagus	6–31 (16)	28–40 (34)	5–33 (19)	19–58 (35)	16	14–19 (16)
Intestinal bifurcation to ventral sucker	118–171 (146)	114–222 (179)	36–117 (82)	103–239 (160)	142	83–111 (105)
Pre-vitelline distance	189–287 (221)	219–330 (262)	126–211 (168)	143–353 (243)	182	111–134 (123)
Vitellarium to ventral sucker	232–318 (268)	255–418 (363)	110–196 (148)	101–254 (168)	147	77–137 (112)
Ventral sucker	120–171 × 124–171 (146 × 147)	148–210 × 156–214 (184 × 189)	98–169 × 102–176 (127 × 133)	88–170 × 101–181 (132 × 148)	105 × 113	58–76 × 65–88 (69 × 76)
Cirrus-sac	226–395 × 81–144 (299 × 110)	307–465 × 135–230 (410 × 172)	174–327 × 55–98 (246 × 76)	186–299 × 63–108 (235 × 85)	188 × 63	143 × 38
Ventral sucker to ovary	27–96 (59)	40–75 (55)	19–75 (38)	54–177 (114)	119	33–59 (48)
Ovary	59–120 × 91–160 (82 × 110)	104–173 × 153–214 (136 × 187)	47–83 × 45–84 (60 × 59)	64–176 × 72–227 (113 × 130)	122 × 115	48–89 × 38–62 (58 × 52)
Ovary to anterior testis	0	0	0–13 (1)	0	0	0–30 (15)
Anterior testis	93–130 × 114–260 (109 × 174)	129–185 × 211–296 (165 × 264)	55–96 × 50–111 (73 × 79)	61–147 × 59–140 (98 × 105)	76 × 58	46–64 × 30–49 (54 × 42)
Distance between testes	0	0	0	0	0	0
Posterior testis	94–165 × 82–233 (131 × 141)	200–268 × 209–271 (226 × 244)	59–113 × 51–122 (87 × 81)	75–182 × 72–156 (111 × 113)	71 × 74	51–64 × 30–60 (57 × 49)
Post-testicular distance	186–257 (229)	153–300 (237)	71–144 (93)	132–283 (209)	207	107–163 (128)
Post-caecal distance	37–93 (68)	65–113 (80)	17–70 (45)	21–86 (52)	49	27–43 (37)
Eggs	49–56 × 27–30 (51 × 29)	50–56 × 27–34 (53 × 30)	42–60 × 26–36 (53 × 32)	50–65 × 25–39 (56 × 32)	54 × 32	48–61 × 24–35 (53 × 31)
Width (%)^a^	39.2–49.8 (43.1)	40.6–46.1 (42.1)	32.5–49.0 (37.5)	31.7–46.5 (36.4)	30.5	31.7–37.9 (34.0)
Forebody (%)^a^	37.5–41.6 (39.7)	37.9–40.3 (39.1)	32.5–44.9 (41.3)	30.5–40.2 (35.5)	33.2	35.3–39.2 (36.7)
Sucker length ratio	1:1.05–1.15 (1.09)	1:0.99–1.14 (1.07)	1:0.96–1.50 (1.24)	1:0.96–1. 67 (1.25)	1:1.33	1:1.06–1.33 (1.17)
Sucker width ratio	1:0.79–0.89 (0.84)	1:0.76–0.87 (0.80)	1:0.82–1.01 (0.88)	1:0.73–1.02 (0.92)	1:0.88	1:0.83–0.97 (0.90)
Oral sucker: pharynx width	1:1.04–1.28 (1.19)	1:1.22–1.35 (1.29)	1:1.45–1.83 (1.62)	1:1.59–2.04 (1.76)	1:1.82	1:1.43–2.05 (1.65)
Ventral sucker to ovary (%)^a^	2.55–6.82 (4.59)	3.16–39.1 (34.4)	2.31–9.24 (4.97)	4.81–13.5 (9.76)	12.0	5.24–8.60 (7.45)
Post-testicular distance (%)^a^	16.7–22.1 (18.7)	12.9–15.6 (14.7)	9.48–16.6 (12.1)	13.4–22.5 (17.9)	20.9	17.2–23.1 (19.9)
Prepharynx (%)^a^	0–2.32 (0.79)	0–1.60 (0.84)	0–5.75 (2.99)	1.26–3.46 (2.50)	3.77	1.43–3.60 (2.15)
Oesophagus (%)^a^	0.48–2.08 (1.25)	1.91–2.42 (2.17)	0.59–4.16 (2.54)	1.84–4.24 (3.06)	1.65	2.14–2.87 (2.56)
Intestinal bifurcation to ventral sucker distance (%)^a^	10.2–13.9 (11.9)	9.60–12.2 (11.1)	4.40–13.1 (10.7)	10.7–16.8 (13.8)	14.3	14.7–17.8 (16.3)
Vitellarium to ventral sucker distance (%)^a^	20.4–22.3 (21.7)	21.3–25.5 (22.7)	15.6–22.6 (19.3)	9.61–17.7 (14.6)	14.9	13.7–19.7 (17.4)
Ovary to anterior testis (%)^a^	0	0	0–1.58 (0.15)	0	0	0–4.35 (2.26)
Distance between testes (%)^a^	0	0	0	0	0	0
Cirrus-sac length (%)^a^	21.7–26.3 (24.1)	24.1–27.1 (25.8)	26.1–39.4 (32.0)	16.3–25.6 (20.5)	19.1	20.3
Pre-vitelline distance (%)^a^	16.0–19.3 (18.0)	13.5–18.5 (16.5)	15.5–27.5 (22.0)	12.8–28.8 (21.0)	18	17.2–22.8 (19.3)
Anterior testis length (%)^a^	7.79–10.7 (8.87)	9.56–11.0 (10.4)	7.61–11.9 (9.50)	6.28–12.2 (8.47)	7.70	6.55–10.4 (8.42)
Posterior testis length (%)^a^	8.07–14.0 (10.7)	13.4–16.9 (14.3)	8.68–13.2 (11.2)	7.89–13.5 (9.50)	7.14	7.64–10.3 (8.88)

^a^%, percent of body length

Testes 2, oval, entire, tandem, in mid-hindbody. External seminal vesicle large, but usually obscured by eggs. Cirrus-sac claviform, mainly dorsal to ventral sucker. Internal seminal vesicle oval. Pars prostatica vesicular. Ejaculatory duct long, muscular. Genital atrium distinct. Genital pore sinistral, at bifurcal level.

Ovary trilobate, immediately pre-testicular, separated from ventral sucker. Laurer’s canal opens dorsally to sinistral part of anterior testis. Seminal receptacle dorsal or dorso-lateral to ovary. Mehlis’ gland dorsal to ovary. Uterus intercaecal, mostly pre-testicular, passes ventrally to ovary, overlaps ventral sucker. Eggs tanned, operculate. Metraterm shorter than cirrus-sac, distal extremity with large folded muscular pad. Vitellarium follicular, reaching from level of mid to anterior pharynx to posterior extremity, confluent or nearly so in forebody and confluent in post-testicular region; lateral and ventral to caeca.

Excretory pore dorsal, in mid post-testicular region; vesicle reaches to testes, not traced further.

### Remarks

This species is characterised by molecular means and distinguished from similar congeners by the following morphological characteristics (Table 5). Molecular results are available only for worms from Palau. The Heron Island worms are treated as the same species here, but some morphometric differences are evident and discussed. *Lepotrema clavatum* has a relatively longer forebody, a less distinct preoral lobe, longer prepharynx and oesophagus (going by the “type-series”), a longer intestinal bifurcation to ventral sucker distance, a larger anterior testis, and relatively smaller pharynx and post-testicular and post-caecal distances. *Lepotrema acanthochromidis* n. sp. has a less distinctive pre-oral lobe, a relatively smaller pharynx, a longer prepharynx, a relatively longer oesophagus and pre-vitelline distance, a relatively shorter cirrus-sac and the gonads tend to be slightly larger. *Lepotrema hemitaurichthydis* n. sp. has a less distinct pre-oral lobe, a relatively longer oesophagus, a relatively shorter pre-vitelline distance, a longer ventral sucker to ovary distance, the gonads are slightly larger, the post-testicular distance is shorter and the caeca are longer. *Lepotrema incisum* has deeply incised testes. *Lepotrema monile* has a small sphincter rather than a strong muscular pad around metraterm.

The Palau and Heron Island specimens differ slightly, with the Palau specimens having a relatively longer pre-oral lobe, shorter oesophagus, longer ventral sucker to ovary distance and smaller gonads. These differences are not of the magnitudes that distinguish this form from other species, so they are considered intraspecific variation here.

We have examined only a single specimen of *M. vidua* at each of Palau and Heron Island, both being infected with multiple specimens of *L. melichthydis* n. sp. Four individuals of *M. vidua* examined in French Polynesia were not infected. In addition, we have examined 344 specimens of 13 species of Balistidae in the Indo-West Pacific region without finding infections of this species. We thus infer that it is oioxenous to *M. vidua*, or potentially stenoxenous to *Melichthys*, which has just three recognised species.

Pritchard (1963) and Yamaguti (1970) reported *L*. *clavatum* from *M. vidua* from Hawaiian waters. Yamaguti (1970) described the worms and illustrated the terminal genital and the “ovarian complex”, but not the whole worm and Pritchard (1963) did not describe the worm. These records may represent *L. melichthydis* n. sp., but using the few measurements supplied by Yamaguti (1970) the oesophagus in the Hawaiian form is much longer than found in any of the worms we have studied. The range of sucker-width ratios derived from the measurements given by Yamaguti (1970) is very large (1:0.80–1.33), whereas in all our specimens the ventral sucker is distinctly smaller than the oral sucker at a ratio of 1:0.76–0.89 There is a clear need for sequencing and morphological study of specimens from a range of Hawaiian fish species.

***Lepotrema amansis*** **n. sp.**

Syn. *Lepotrema clavatum* Bray & Cribb (1996c) in part

*Type-host*: *Amanses scopas* (Cuvier) (Tetraodontiformes: Monacanthidae), broom filefish.

*Type-locality*: Off Heron Island (23°27′S, 151°55′E), Great Barrier Reef, Australia.

*Type-material*: Holotype (QM G237503); paratypes (QM G237504–20; NHMUK 2018.7.23.18–22).

*Site in host*: Intestine.

*Prevalence*: In 5 of 7 fish examined.

*Representative DNA sequences*: ITS2 rDNA, four identical replicates (one submitted to GenBank MH730001); *cox*1 mtDNA, four replicates (all submitted to GenBank MH730029–32); 28S rDNA, one sequence (submitted to GenBank MH730016).

*ZooBank registration*: To comply with the regulations set out in article 8.5 of the amended 2012 version of the *International Code of Zoological Nomenclature* (ICZN, 2012), details of the new species have been submitted to ZooBank. The Life Science Identifier (LSID) for *Lepotrema amansis* n. sp. is urn:lsid:zoobank.org:act:0509A1E0-EAE4-478F-99A1-69B20E7C3B32.

*Etymology*: The specific epithet is derived from the generic name of the host species.

### Description (Fig. 17)

[Based on 26 whole-mounted specimens; measurements in Table 5.] Body oblong. Tegument finely spined, spines reaching close to posterior extremity. Oral sucker large, broadly oval, subterminal. Ventral sucker oval, of similar length to, but mostly narrower than oral sucker, equatorial. Prepharynx short, thick-walled. Pharynx large, oval. Oesophagus short, narrow. Intestinal bifurcation just in posterior forebody. Caeca broad, reach to about middle of post-testicular region.

Testes 2, oval, entire, virtually tandem or slightly oblique, in mid hindbody. External seminal vesicle oval, often obscured by eggs. Cirrus-sac large, claviform, mainly dorsal to ventral sucker, reaching to ovary. Internal seminal vesicle rounded. Pars prostatica vesicular. Ejaculatory duct long, muscular. Genital atrium distinct. Genital pore sinistral, at bifurcal level.

Ovary strongly or weakly trilobate or more or less globular, immediately pre-testicular, separated from ventral sucker. Laurer’s canal opens dorsally, sinistrally to anterior testis, sometimes dorsal to left caecum. Seminal receptacle dorsal or dorso-lateral to ovary. Mehlis’ gland dorsal to ovary. Uterus intercaecal, mostly pre-testicular, passes ventrally to ovary, overlaps ventral sucker. Eggs tanned, operculate. Metraterm shorter than cirrus-sac, distal extremity with large folded muscular pad. Vitellarium follicular, follicles sparse, reaching from pharynx to posterior extremity, confluent in post-testicular region; lateral and ventral to caeca.

Excretory pore dorsal, in posterior post-testicular region; vesicle reaches to ovary.

### Remarks

This species is characterised by molecular means (Table 1) and distinguished from similar congeners by the following morphological characteristics (Table 5). *Lepotrema clavatum* is distinctly larger with mainly larger features, a longer prepharynx and oesophagus (cf. “type-series”), relatively smaller suckers and pharynx, relatively larger testes and relatively shorter cirrus-sac and post-testicular and post-caecal distances. *Lepotrema acanthochromidis* n. sp. is usually distinctly larger, with a longer pre-oral lobe, relatively slightly smaller suckers and pharynx, a longer oesophagus, a relatively slightly shorter cirrus-sac and larger gonads. *Lepotrema hemitaurichthydis* n. sp. is usually distinctly larger and broader, with a tendency to have a longer oesophagus, a longer pre-vitelline distance, a relatively slightly shorter cirrus-sac, larger testes and longer caeca. *Lepotrema incisum* has deeply incised testes. *Lepotrema melichthydis* n. sp. is distinctly larger, with a distinctly longer pre-oral lobe, a relatively shorter prepharynx, relatively smaller suckers, a relatively shorter cirrus-sac and a relatively longer post-testicular distance. *Lepotrema monile* has a small sphincter rather than a strong muscular pad around metraterm.

We have examined nine individuals of *Amanses scopas* on the Great Barrier Reef; five of these were infected with *L. amansis* n. sp. No infection of this or any other species of *Lepotrema* has been seen by us in 130 individuals of 28 other species of Monacanthidae examined in the region, apart from the records of *L. canthescheniae* in the southern Great Barrier Reef/ New South Wales endemic fish *Cantheschenia grandisquamis*.

***Lepotrema cirripectis*** **n. sp.**

*Type-host*: *Cirripectes filamentosus* (Alleyne & Macleay) (Perciformes: Blenniidae), filamentous blenny.

*Other hosts*: *Cirripectes chelomatus* Williams & Maugé, Lady Musgrave blenny; *Cirripectes stigmaticus* Strasburg & Schultz, red-streaked blenny (both Blenniidae).

*Type-locality*: Off Lizard Island (14°40′S, 145°28′E), Great Barrier Reef, Australia.

*Other locality*: Off Heron Island (23°27′S, 151°55′E), Great Barrier Reef, Australia.

*Type-material*: Holotype (QM G237521); paratypes: ex *C. filamentosus* (QM G237522–35, 42–50; NHMUK 2018.7.23.23–32); ex *C. chelomatus* (QM G237536–41; NHMUK 2018.7.23.28–29); ex *C. stigmaticus* (QM G237551).

*Site in host*: Intestine.

*Prevalence*: Ex *C. filamentosus* (in 7 of 8 fish examined); ex *C. stigmaticus* (in 2 of 2 fish examined); ex *C. chelomatus* (in 4 of 4 fish examined).

*Representative DNA sequences*: ITS2 rDNA, six identical replicates (two submitted to GenBank MH730004–05); *cox*1 mtDNA, six replicates (all submitted to GenBank MH730036–41); 28S rDNA, one sequence (submitted to GenBank MH730018).

*ZooBank registration*: To comply with the regulations set out in article 8.5 of the amended 2012 version of the *International Code of Zoological Nomenclature* (ICZN, 2012), details of the new species have been submitted to ZooBank. The Life Science Identifier (LSID) for *Lepotrema cirripectis* n. sp. is urn:lsid:zoobank.org:act:1F4D160D-6439-4558-B106-17E9D5026830.

*Etymology*: The specific epithet is derived from the generic name of the host species.

### Description (Figs. 18–21)

[Based on 40 whole-mounted specimens, 32 from off Lizard Island (31 ex *C. filamentosus* and 1 ex *C. stigmaticus*) and 8 from off Heron Island (all in *C. chelomatus*); measurements in Table 5.] Body elongate-oval. Tegument finely spined in forebody. Oral sucker oval, subterminal. Ventral sucker rounded, longer than, but of similar width to, oral sucker, pre-equatorial. Prepharynx distinct. Pharynx oval. Oesophagus distinct, narrow. Intestinal bifurcation in mid to posterior forebody. Caeca broad, reach close to posterior extremity.

Testes 2, small, oval, entire, virtually tandem to slightly oblique, in mid hindbody. Post-testicular region long. External seminal vesicle oval, but usually obscured by eggs. Cirrus-sac claviform, mainly dorsal to ventral sucker. Internal seminal vesicle oval to rounded. Pars prostatica vesicular. Ejaculatory duct long, muscular. Genital atrium distinct. Genital pore sinistral, at level of the anterior margin of the ventral sucker or just anterior.

Ovary trilobate, immediately pre-testicular, distinctly separated from ventral sucker. Laurer’s canal opens dorsally to anterior testis. Seminal receptacle dorsal or dorso-lateral to ovary. Mehlis’ gland dorsal to ovary. Uterus intercaecal, pre-testicular, overlaps ovary, little or no overlap of ventral sucker. Eggs tanned, operculate. Metraterm shorter than cirrus-sac, distal extremity with folded muscular pad. Vitellarium follicular, reaching from bifurcal level to posterior extremity, confluent in forebody and post-testicular region; lateral and to caeca.

Excretory pore dorsal, in anterior post-testicular region; vesicle reaches to at least ovary.

### Remarks

This species is characterised by molecular means (Table 1) and distinguished from similar congeners by the following morphological characteristics (Table 5). *Lepotrema clavatum* is larger with mainly larger characters, including the oral sucker, pharynx and testes, it has a longer forebody, a distinct pre-oral lobe (in examined specimens), a shorter pre-vitelline distance, a relatively longer cirrus-sac, and shorter ventral sucker to ovary, post-testicular and post-caecal distances. *Lepotrema acanthochromidis* n. sp. has a longer pre-oral lobe, a shorter ventral sucker to ovary distance and larger testes. *Lepotrema adlardi* is narrower, particularly in the forebody, the forebody is longer, the pre-oral lobe is distinct, it has longer prepharynx and pharynx, the intestinal bifurcation is more posterior, the extension of the vitellarium into forebody is much shorter and the ventral sucker to ovary distance is longer. *Lepotrema amansis* n. sp. has relatively larger suckers and pharynx, a longer cirrus-sac and shorter ventral sucker to ovary and post-testicular distances. *Lepotrema hemitaurichthydis* n. sp. is broader, with a longer forebody and pre-oral lobe, slightly larger suckers, a larger pharynx, a shorter ventral sucker to ovary distance, larger testes and a shorter post-testicular distance. *Lepotrema incisum* has deeply incised testes. *Lepotrema melichthydis* n. sp. has a distinct pre-oral lobe, a relatively shorter prepharynx and oesophagus, a larger pharynx, shorter pre-vitelline and ventral sucker to ovary distances and a relatively smaller ovary. *Lepotrema monile* has a small sphincter rather than a strong muscular pad around metraterm.

*Lepotrema cirripectis* n. sp. has been detected in five of 11 individuals of two species of *Cirripectes* examined at Lizard Island and in one species of *Cirripectes* examined at Heron Island. No specimens relating to *Lepotrema* have been found by us in 240 individuals of 28 species of other blenniid genera from the region, allowing the inference that this species is stenoxenous for the genus *Cirripectes*. Heron Island specimens are all smaller than the Lizard Island specimens, but with similar proportions.

***Lepotrema justinei*** **n. sp.**

Syn. *Lepotrema* cf. *clavatum* of Bray & Justine (2012)

*Type-host*: *Sufflamen fraenatum* (Latreille) (Tetraodontiformes: Balistidae), masked triggerfish.

*Type-locality*: Interior Lagoon near Recif Toombo (22°33′S, 166°29′E), New Caledonia.

*Other localities*: Inside Lagoon, facing Recif Toombo (22°32′S, 166°27′E), Interior Lagoon near Recif Toombo (22°33′S, 166°29′E), New Caledonia.

*Type-material*: Holotype (MNHN JNC2772Aa); paratypes (MNHN JNC2372; JNC2763, JNC2772Ab; BMNH 2012.5.25.18).

*Site in host*: Intestine.

*ZooBank registration*: To comply with the regulations set out in article 8.5 of the amended 2012 version of the *International Code of Zoological Nomenclature* (ICZN, 2012), details of the new species have been submitted to ZooBank. The Life Science Identifier (LSID) for *Lepotrema justinei* n. sp. is urn:lsid:zoobank.org:act:9D10C114-06F7-418C-B695-68EEC8BA09A9.

*Etymology*: The species is name after Professor Jean-Lou Justine of the Muséum National d’Histoire Naturelle, Paris, France, in recognition of his massive contributions to marine fish parasitology.

### Description (Fig. 22)

[Based on 6 whole-mounted specimens; measurements in Table 6.] Body pyriform, widest in hindbody. Tegumental spines reaching close to posterior extremity. Eye-spot pigment scattered around pharynx and oral sucker regions in some, but not all, specimens. Oral sucker large, subglobular, subterminal. Ventral sucker oval, of similar size to oral sucker, just pre-equatorial. Prepharynx short, in posterior cavity of oral sucker, thick-walled. Pharynx large, oval. Oesophagus short, narrow. Intestinal bifurcation in posterior forebody. Caeca broad, reach to about middle of post-testicular region.

**Figs. 22–28:**
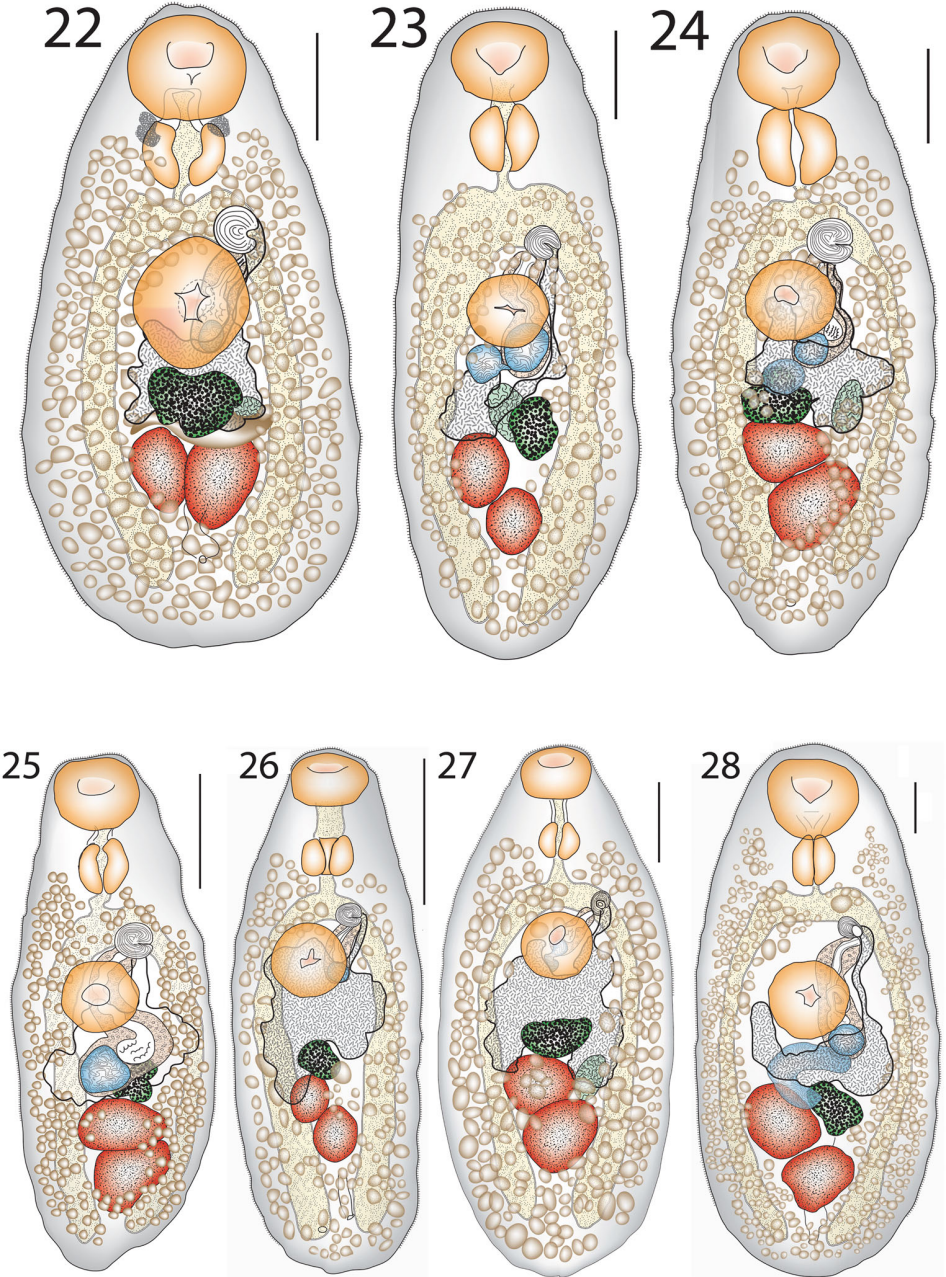
*Lepotrema* spp. 22, *Lepotrema justinei* n. sp. ex *Sufflamen fraenatum*, New Caledonia, holotype, ventral view (redrawn from Bray & Justine, 2012); 23–25, *Lepotrema moretonense* n. sp.; 23, ex *Prionurus microlepidotus*, off Amity, North Stradbroke Island, holotype, ventral view; 24, ex *Prionurus maculatus*, Moreton Bay, ventral view; 25, ex *Selenotoca multifasciata*, Moreton Bay, ventral view. 26, 27, *Lepotrema amblyglyphidodonis* n. sp.; 26, ex *Amblyglyphidodon curacao*, off Heron Island, holotype, ventral view; 27, ex *Amphiprion akyndynos*, off Heron Island, ventral view; 28, *Lepotrema canthescheniae* Bray & Cribb, 1996 ex *Cantheschenia grandisquamis*, off Heron Island, ventral view (redrawn from Bray & Cribb, 1996c). *Scale-bars*: 200 μm

**Table 6:** Dimensions of *Lepotrema justinei* n. sp., *L. moretonense* n. sp. and *L. canthescheniae* Bray & Cribb, 1996

Species	*Lepotrema justinei*	*Lepotrema moretonense*			*Lepotrema canthescheniae*
Host	*Sufflamen fraenatum*	*Prionurus microlepidotus*	*Prionurus maculatus*	*Selenotoca multifasciata*	*Cantheschenia grandisquamis*
Locality	New Caledonia	Moreton Bay			Heron Island
n	6	8	5	1	4
Body	722–1,326 × 460–681 (1,096 × 552)	1,061–1,474 × 325–525 (1,270 × 477)	1,138–1,271 × 438–586 (1,211 × 500)	918 × 361	1,813–2,036 × 629–859 (1,948 × 703)
Forebody	307–483 (399)	400–595 (499)	455–530 (490)	358	734–795 (779)
Pre-oral lobe	17–39 (26)	10–28 (20)	10–20 (14)	9	52–63 (57)
Oral sucker	100–214 × 111–240 (165 × 180)	104–184 × 147–221 (168 × 199)	167–198 × 199–231 (183 × 210)	133 × 153	267–289 × 309–318 (275 × 314)
Prepharynx	0–17 (3)	0–35 (7)	0	0	0
Pharynx	76–176 × 75–172 (133 × 122)	94–169 × 88–160 (146 × 143)	142–185 × 143–170 (164 × 159)	114 × 87	193–219 × 162–188 (204 × 176)
Oesophagus	7–35 (23)	9–47 (30)	12–44 (23)	31	39–64 (52)
Intestinal bifurcation to ventral sucker	54–103 (80)	99–196 (148)	107–139 (118)	91	174–238 (208)
Pre-vitelline distance	107–247 (194)	225–393 (296)	279–361 (315)	205	290–303 (295)
Vitellarium to ventral sucker	167–236 (204)	134–255 (204)	136–200 (175)	154	431–502 (483)
Ventral sucker	109–264 × 98–240 (201 × 172)	114–169 × 125–184 (156 × 170)	165–188 × 171–192 (177 × 181)	130 × 141	283–290 × 270–290 (288 × 278)
Cirrus-sac	97–268 × 30–83 (195 × 58)	243–367 × 72–106 (299 × 87)	260–326 × 85–97 (293 × 92)	328 × 70	438–464 × 156–171 (449 × 165)
Ventral sucker to ovary	0–7 (1)	37–116 (81)	33–74 (49)	33	52–147 (94)
Ovary	97–141 × 82–192 (122 × 142)	53–136 × 79–124 (98 × 106)	77–123 × 84–146 (102 × 118)	88 × 89	204–213 × 190–226 (209 × 212)
Ovary to anterior testis	0	0–40 (5)	0	0	0
Anterior testis	171–255 × 99–167 (194 × 130)	103–166 × 130–184 (141 × 152)	113–127 × 131–195 (120 × 159)	105 × 143	263–290 × 247–259 (271 × 253)
Distance between testes	0	0	0	0	0
Posterior testis	133–251 × 94–185 (193 × 130)	148–200 × 125–174 (166 × 150)	157–174 × 137–181 (167 × 154)	138 × 137	258–351 × 251–316 (323 × 269)
Post-testicular distance	129–237 (185)	148–233 (187)	102–197 (165)	115	213–232 (227)
Post-caecal distance	50-120 (91)	39-100 (75)	46-163 (95)	22	102-132 (114)
Eggs	44–62 × 27–33 (57 × 31)	50–58 × 23–31 (54 × 28)	46–58 × 29–34 (54 × 32)	52 × 29	56–67 × 25–30 (61 × 28)
Width (%)^a^	43.2–71.0 (53.1)	30.5–42.9 (37.6)	37.3–46.7 (41.3)	39.3	30.9–43.8 (36.2)
Forebody (%)^a^	32.7–42.6 (37.8)	37.6–42.8 (39.3)	38.3–44.4 (40.5)	39.0	39.0–40.6 (40.0)
Sucker length ratio	1:1.05–1.31 (1.19)	1:0.89–1.10 (0.94)	1:0.92–1.03 (0.97)	1:0.98	1:1.00–1.07 (1.05)
Sucker width ratio	1:0.86–1.06 (0.94)	1:0.82–0.89 (0.85)	1:0.83–0.89 (0.86)	1:0.92	1:0.87–0.91 (0.88)
Oral sucker: pharynx width	1:1.36–1.73 (1.48)	1:1.28–1.68 (1.41)	1:1.24–1.43 (1.32)	1:1.76	1:1.64–1.96 (1.78)
Ventral sucker to ovary (%)^a^	0–0.53 (0.09)	3.53–8.98 (6.27)	2.66–6.39 (4.07)	3.62	2.87–7.50 (4.78)
Post-testicular distance (%)^a^	15.9–20.2 (17.5)	12.9–16.9 (14.7)	8.98–16.0 (13.5)	12.6	11.3–11.8 (11.6)
Prepharynx (%)^a^	0–2.36 (0.39)	0–3.29 (0.60)	0	0	0
Oesophagus (%)^a^	0.85–3.13 (2.18)	0.71–3.99 (2.35)	1.06–3.86 (1.95)	3.35	1.99–3.23 (2.68)
Intestinal bifurcation to ventral sucker distance (%)^a^	4.52–10.7 (7.82)	8.79–15.1 (11.6)	8.72–10.9 (9.76)	9.89	9.60–12.1 (10.7)
Vitellarium to ventral sucker distance (%)^a^	14.0–27.7 (19.9)	12.6–18.6 (16.0)	11.7–15.9 (14.5)	16.7	23.8–25.6 (24.8)
Ovary to anterior testis (%)^a^	0	0–2.72 (0.34)	0	0	0
Distance between testes (%)^a^	0	0	0	0	0
Cirrus-sac length (%)^a^	13.5–20.8 (17.7)	19.9–26.9 (23.6)	21.1–26.6 (24.3)	35.7	22.1–24.2 (23.1)
Pre-vitelline distance (%)^a^	14.9–19.6 (17.9)	20.3–27.5 (23.3)	22.7–28.7 (26.1)	22.3	14.5–16.7 (15.2)
Anterior testis length (%)^a^	14.4–23.7 (18.7)	9.62–12.1 (11.0)	9.03–10.4 (10.0)	11.4	13.1–14.8 (13.9)
Posterior testis length (%)^a^	15.7–19.9 (18.1)	10.4–14.7 (13.1)	12.7–15.0 (13.8)	15.1	13.2–18.4 (16.6)

^a^%, percent of body length

Testes 2, oval, entire or slightly irregular, symmetrical or slightly oblique, in mid hindbody. External seminal vesicle large, but usually obscured by eggs. Cirrus-sac claviform, mainly dorsal to ventral sucker. Internal seminal vesicle oval. Pars prostatica vesicular. Ejaculatory duct long, thick-walled. Genital atrium distinct. Genital pore sinistral, ventral to sinistral caecum, at about level of anterior margin of the ventral sucker.

Ovary trilobate, immediately pre-testicular, close to ventral sucker. Laurer’s canal not detected. Seminal receptacle dorsal or dorso-lateral to ovary. Mehlis’ gland dorsal to ovary. Uterus intercaecal, pre-testicular, passes ventrally to ovary, overlaps posterior edge of ventral sucker. Eggs tanned, operculate. Metraterm shorter than cirrus-sac, distal extremity with large folded muscular pad. Vitellarium follicular, reaching from anterior part of pharynx to posterior extremity, confluent in forebody and post-testicular region; lateral and ventral, but not dorsal to caeca.

Excretory pore dorsal, in anterior post-testicular region; vesicle reaches to testes, not traced further.

### Remarks

This species is not characterised by molecular means but is distinguished from similar congeners by the following morphological characteristics (Table 6).

It is the only species with more or less symmetrical testes, and, probably as a result, it tends to be broader than the other species. *Lepotrema clavatum* is a larger worm but is relatively slightly narrower, it has a longer forebody, a less distinct pre-oral lobe, a longer prepharynx (cf. “type series”), a relatively smaller pharynx, a relatively longer oesophagus (cf. “type series”), a more anterior intestinal bifurcation and vitelline extent, a relatively smaller ventral sucker, a longer ventral sucker to ovary distance, a shorter post-testicular distance and longer caeca. *Lepotrema acanthochromidis* n. sp. has smaller suckers, a longer prepharynx, a smaller pharynx, a longer oesophagus, a more anterior intestinal bifurcation, the ovary is separated from the ventral sucker and the gonads are smaller. *Lepotrema amansis* n. sp. is distinctly narrower, with a shorter pre-oral lobe, a longer prepharynx, a relatively longer cirrus-sac, a longer ventral sucker to ovary distance, relatively distinctly smaller gonads and a shorter post-testicular distance. *Lepotrema cirripectis* n. sp. is narrower, with a shorter pre-oral lobe, a longer prepharynx, a shorter pharynx, longer intestinal bifurcation to ventral sucker and ventral sucker to ovary distances, smaller testes and longer caeca. *Lepotrema hemitaurichthydis* n. sp. has a shorter pre-oral lobe, smaller suckers, longer prepharynx and cirrus-sac, the ovary separated from the ventral sucker, the gonads are smaller, the post-testicular region is shorter, and the caeca are longer. *Lepotrema incisum* has deeply incised testes. *Lepotrema melichthydis* n. sp. has a slightly longer pre-oral lobe, slightly smaller suckers, a shorter pre-vitelline distance, a longer ventral sucker to ovary distance and relatively slightly smaller gonads. *Lepotrema monile* has a small sphincter rather than a strong muscular pad around metraterm.

*Lepotrema justinei* n. sp. has been found only in *S. fraenatum* from off New Caledonia, where three of 14 fish examined were infected. We have not seen this species in eight *S. fraenatum* examined from off Heron Island or in seven examined at Ningaloo Reef. In addition, examination of a further 167 individuals of *S. bursa* (Bloch & Schneider, 1801) and *S. chrysopterum* from the Indo-West Pacific region (together with many other balistids) have not been infected with this species. The single specimen reported as *Lepotrema clavatum* from *S. chrysopterum* by Bray & Cribb (1996) from off Heron Island is clearly distinct (see below as *Lepotrema* sp. 3).

***Lepotrema moretonense*** **n. sp.**

*Type-host*: *Prionurus microlepidotus* Lacépède (Perciformes: Acanthuridae), sixplate sawtail

*Other hosts*: *Prionurus maculatus* Ogilby (Acanthuridae), yellowspotted sawtail; *Selenotoca multifasciata* (Richardson) (Perciformes: Scatophagidae), spotbanded scat.

*Type-locality*: Off Amity (27°24′S, 153°26′E), North Stradbroke Island, Queensland, Australia.

*Other localities*: *P. maculatus*: off Amity, North Stradbroke Island, Queensland; *S. multifasciata*, off Green Island, Moreton Bay (27°25′S, 153°14′E), Australia.

*Type-material*: Holotype (QM G237552); paratypes: ex *P. microlepidotus* (QM G237553–7; NHMUK 018.7.23.33–34); ex *P. maculatus* (QM G237558–61; NHMUK 2018.7.23.35–36); ex *S. multifasciata* (QM G237562).

*Site in host*: Intestine.

*Prevalence*: Ex *P. microlepidotus* (in 8 of 8 fish examined); ex *P. maculatus* (in 2 of 2 fish examined); ex *S. multifasciata* (in 2 of 36 fish examined).

*Representative DNA sequences*: ITS2 rDNA, six identical replicates (three submitted to GenBank MH730011–13); *cox*1 mtDNA, five replicates (all submitted to GenBank MH730051–55); 28S rDNA, two identical replicates (one submitted to GenBank MH730023).

*ZooBank registration*: To comply with the regulations set out in article 8.5 of the amended 2012 version of the *International Code of Zoological Nomenclature* (ICZN, 2012), details of the new species have been submitted to ZooBank. The Life Science Identifier (LSID) for *Lepotrema moretonense* n. sp. is urn:lsid:zoobank.org:act:02130E56-E6D9-4B85-9081-DF9675E5F74D.

*Etymology*: The specific epithet is derived from the locality from where this species is described.

### Description (Figs. 23–25)

[Based on 14 whole-mounted specimens (including a hologenophore); measurements in Table 6.] Body elongate-oval. Tegument finely spined, spines reaching to about level of posterior testis. Oral sucker large, subglobular, subterminal. Ventral sucker oval, of similar size to oral sucker, just pre-equatorial. Prepharynx short, in posterior cavity of oral sucker, thick-walled. Pharynx large, oval. Oesophagus short, narrow. Intestinal bifurcation in mid-forebody. Caeca broad, reach close to posterior extremity.

Testes 2, oval, entire, virtually tandem or slightly oblique, in mid-hindbody. External seminal vesicle saccular, often obscured by eggs. Cirrus-sac claviform, sigmoid or flexed, mainly dorsal to ventral sucker but reaches distinctly into hindbody. Internal seminal vesicle oval. Pars prostatica vesicular. Ejaculatory duct long, thick-walled. Genital atrium distinct. Genital pore sinistral, ventral to sinistral caecum, distinctly anterior to ventral sucker, may be at bifurcal level.

Ovary trilobate, immediately pre-testicular, separated from ventral sucker. Laurer’s canal opens dorsally to anterior part of anterior testis, not easily detected. Seminal receptacle dorsal or dorso-lateral to ovary. Mehlis’ gland dorsal to ovary. Uterus intercaecal, pre-testicular, passes ventrally to ovary. Eggs tanned, operculate. Metraterm shorter than cirrus-sac, distal extremity with large folded muscular pad. Vitellarium follicular, reaching from level of posterior part of pharynx, oesophagus or intestinal bifurcation to posterior extremity, confluent in forebody and post-testicular region; lateral and ventral, but not dorsal to caeca.

Excretory pore dorsal, in anterior post-testicular region or at level of caecal ends; vesicle reaches to testes, not traced further.

### Remarks

This species is characterised by molecular means (Table 1) and distinguished from similar congeners by the following morphological characteristics (Table 6). *Lepotrema clavatum* is larger with a slightly longer forebody, a shorter pre-oral lobe, longer prepharynx (cf. “type-series”) and oesophagus, a shorter pre-vitelline distance, a longer cirrus-sac, a relatively shorter ventral sucker to ovary distance, slightly larger testes, a shorter post-testicular distance and longer caeca. *Lepotrema acanthochromidis* n. sp. is very similar but the prepharynx tends to be longer, and there is a tendency for a slightly shorter pharynx and ventral sucker to ovary distance. *Lepotrema adlardi* is slightly narrower, with a distinctly narrower forebody, a smaller oral sucker, a longer prepharynx, a shorter intestinal bifurcation to ventral sucker distance, a much longer pre-vitelline distance and a slightly shorter cirrus-sac and ventral sucker to ovary distance. *Lepotrema amansis* n. sp. is smaller, but relatively slightly narrower, it has a less distinct pre-oral lobe, a slightly shorter pre-vitelline distance, a shorter ventral sucker to ovary distance and a smaller posterior testis. *Lepotrema cirripectis* n. sp. has a less distinct pre-oral lobe, slightly smaller suckers, a longer prepharynx, a smaller pharynx, the ventral sucker to ovary distance tends to be longer, the testes tend to be smaller as does the cirrus-sac but the post-testicular distance may be slightly larger. *Lepotrema hemitaurichthydis* n. sp. is very similar, but is wider, with possibly a slightly longer prepharynx, possibly a slightly longer oesophagus, a longer ventral sucker to ovary distance and slightly longer caeca. *Lepotrema justinei* n. sp. is broader, with a relatively slightly larger ventral sucker, a shorter pre-bifurcal distance, a distinct separation of ventral sucker and ovary, symmetrical testes and smaller gonads. *Lepotrema melichthydis* n. sp. has a more prominent pre-oral lobe, a shorter pre-vitelline distance and a slight tendency for the ventral sucker to ovary distance to be smaller.

Numerous specimens of *Lepotrema moretonense* n. sp. have been found in all seven *P. microlepidotus* and both *P. maculatus* examined from Moreton Bay. In addition, a single gravid adult and a single immature specimen (that was sequenced for the ITS2 and *cox*1 datasets) have been found in two of the 36 individuals of *Selenotoca multifasciata* (Scatophagidae) examined from Moreton Bay. We think that these data suggest that this species is effectively stenoxenous to the genus *Prionurus.* We have only examined a single uninfected individual of a *Prionurus* species elsewhere and so cannot comment on the geographical distribution of this species.

***Lepotrema amblyglyphidodonis*** **n. sp.**

Syn. *Lepocreadium* sp. of Bray et al. (1993) and Barker et al. (1994)

*Type-host*: *Amblyglyphidodon curacao* (Bloch) (Perciformes: Pomacentridae), staghorn damselfish.

*Other host*: *Amphipron akyndynos* Allen, Barrier Reef anemonefish (Pomacentridae).

*Type-locality*: Off Heron Island (23°27′S, 151°55′E), Great Barrier Reef, Australia.

*Type-material*: Holotype (QM G237563); paratypes: ex *A. curacao* (QM GL 14775–6); ex *A. akyndynos* (QM G237564).

*Site in host*: Intestine.

*Prevalence*: Ex *A. curacao* (in 5 of 71 fish examined); ex *A. akyndynos* (in 1 of 7 fish examined).

*Representative DNA sequences*: ITS2 rDNA, three identical replicates (two submitted to GenBank MH730002–03); *cox*1 mtDNA, three identical replicates (all submitted to GenBank MH730033–35); 28S rDNA, one sequence (submitted to GenBank MH730017).

*ZooBank registration*: To comply with the regulations set out in article 8.5 of the amended 2012 version of the *International Code of Zoological Nomenclature* (ICZN, 2012), details of the new species have been submitted to ZooBank. The Life Science Identifier (LSID) for *Lepotrema amblyglyphidodonis* n. sp. is urn:lsid:zoobank.org:act:6BF7C15D-FBF0-4650-A5B4-626CD087A97E.

*Etymology*: The specific epithet is derived from the generic name of the type-host.

### Description (Figs. 26–27)

[Based on 3 specimens ex *Amblyglyphidodon curacao* and 1 ex *Amphiprion akyndynos*; measurements in Table 7.] Body elongate-oval. Tegument finely spined, spines reaching close to posterior extremity. Oral sucker large, broadly oval, just subterminal. Ventral sucker oval, of similar size to oral sucker, pre-equatorial. Prepharynx distinct. Pharynx large, oval. Oesophagus short, narrow. Intestinal bifurcation just in posterior forebody. Caeca broad, reach to posterior part of post-testicular region.

**Table 7:** Dimensions of *Lepotrema amblyglyphidodonis* n. sp. and *L. monile* Bray & Cribb, 1998

Species	*Lepotrema amblyglyphidodonis*		*Lepotrema monile*			
Host	*Amblyglyphidodon curacao*	*Amphiprion akyndynos*	*Pomacentrus wardi*	*Pomacentrus amboinensis*	*Pomacentrus chrysurus*	*Stegastes apicalis*
Locality	Heron Island	Heron Island	Heron Island	Lizard Island	Lizard Island	Heron Island
n	3	1	3	2	1	1
Body	685–1,367 × 258–559 (970 × 390)	1,319 × 593	1,002–1,043 × 384–415 (1,020 × 397)	774–936 × 380–400	964 × 367	1,017 × 456
Forebody	228–478 (344)	415	346–354 (351)	285–271	300	350
Pre-oral lobe	0–7 (2)	0	9–13 (11)	6–14	3	0
Oral sucker	72–154 × 103–167 (112 × 139)	130 × 178	111–116 × 141–148 (114 × 144)	95–99 × 118–146	90 × 146	130 × 170
Prepharynx	29–87 (53)	53	25–43 (36)	19–42	44	15
Pharynx	55–113 × 70–85 (84 × 78)	96 × 95	71–90 × 72–97 (83 × 81)	70–78 × 64–80	75 × 78	105 × 130
Oesophagus	22–73 (43)	56	14–24 (20)	19–38	42	15
Intestinal bifurcation to ventral sucker	42–87 (60)	75	77–96 (85)	24–55	35	79
Pre-vitelline distance	135–230 (188)	228	207–354 (305)	175–190	207	203
Vitellarium to ventral sucker	93–248 (156)	187	140	95–96	93	146
Ventral sucker	97–168 × 100–171 (131 × 137)	163 × 183	144–155 × 148–155 (149 × 151)	112–118 × 111–125	126 × 131	179 × 190
Cirrus-sac	99–135 × 38–55 (112 × 48)	157 × 65	96–126 × 39–45 (113 × 42)	113–147 × 35–40	?	147 × 46
Ventral sucker to ovary	3–80 (49)	102	70–76 (73)	25–121	111	35
Ovary	52–106 × 48–126 (78 × 92)	96 × 169	106–122 × 78–90 (111 × 83)	70–82 × 61–80	90 × 81	91 × 104
Ovary to anterior testis	0	0	0	0	0	0
Anterior testis	62–109 × 57–116 (89 × 96)	149 × 181	95–116 × 74–90 (105 × 80)	87–92 × 83–84	89 × 85	98 × 83
Distance between testes	0	0	0	0	0	0
Posterior testis	72–148 × 58–150 (109 × 111)	173 × 174	105–115 × 79–97 (110 × 89)	93–100 × 80–88	100 × 95	112 × 81
Post-testicular distance	145–260 (192)	255	186–200 (191)	147–183	180	169
Post-caecal distance	44–122 (79)	111	59–71 (63)	67–63	61	53
Eggs	51–61 × 26–42 (56 × 34)	62 × 29	59–63 × 29–30 (61 × 29)	65–68 × 30–33	65 × 36	67 × 31
Width (%)^a^	37.6–41.1 (39.9)	44.9	38.3–39.8 (38.9)	42.8–49.2	38.1	44.8
Forebody (%)^a^	33.3–38.0 (35.4)	31.5	33.8–35.3 (34.4)	29.0–36.8	31.1	34.4
Sucker length ratio	1:1.09–1.34 (1.20)	1:1.26	1:1.30–1.34 (1.31)	1:1.13–1.24	1:1.40	1:1.37
Sucker width ratio	1:0.95–1.02 (0.98)	1:1.03	1:1.04–1.06 (1.05)	1:0.85–0.95	1:0.90	1:1.12
Oral sucker: pharynx width	1:1.48–1.96 (1.77)	1:1.87	1:1.53–1.97 (1.80)	1:1.83–1.84	1:1.88	1:1.30
Ventral sucker to ovary (%)^a^	0.35–9.31 (5.17)	7.70	6.71–7.47 (7.12)	3.23–12.9	11.5	3.40
Post-testicular distance (%)^a^	19.0–21.2 (20.1)	19.3	18.5–19.2 (18.7)	19.0–19.6	18.7	16.6
Prepharynx (%)^a^	3.38–6.36 (5.30)	3.99	2.40–4.25 (3.55)	2.47–4.54	4.62	1.51
Oesophagus (%)^a^	3.17–5.34 (4.19)	4.24	1.34–24.0 (1.94)	2.42–4.03	4.37	1.45
Intestinal bifurcation to ventral sucker distance (%)^a^	5.94–6.36 (6.15)	5.65	7.68–9.20 (8.35)	2.59–7.17	3.68	7.80
Vitellarium to ventral sucker distance	13.5–18.1 (15.5)	14.2	13.8	10.3–12.3	9.64	14.4
Ovary to anterior testis (%)^a^	0	0	0	0	0	0
Distance between testes (%)^a^	0	0	0	0	0	0
Cirrus-sac length (%)^a^	9.88–14.4 (12.1)	11.9	9.44–12.1 (11.0)	14.6–15.7	?	14.5
Pre-vitelline distance (%)^a^	16.8–23.2 (19.9)	17.3	20.4–35.3 (29.9)	18.7–24.5	21.5	20.0
Anterior testis length (%)^a^	7.97–11.1 (9.38)	11.3	9.48–11.1 (10.3)	9.88–11.2	9.20	9.02
Posterior testis length (%)^a^	10.6–12.6 (11.3)	13.1	10.4–11.0 (10.8)	10.7–12.0	10.4	11.0

^a^%, percent of body length

Testes 2, oval, entire, oblique, in mid-hindbody. External seminal vesicle oval. Cirrus-sac small, claviform, mainly dorsal to ventral sucker, not reaching into hindbody. Internal seminal vesicle oval. Pars prostatica vesicular. Ejaculatory duct long, muscular. Genital atrium distinct. Genital pore sinistral, at bifurcal level.

Ovary weakly trilobate, immediately pre-testicular, separated from ventral sucker. Laurer’s canal opens dorsal to left caecum. Seminal receptacle postero-dorsal to ovary, postero-sinistral to anterior testis. Mehlis’ gland dorsal to ovary. Uterus overlaps caeca laterally, overlaps anterior testis, passes ventrally to ovary, overlaps ventral sucker. Eggs tanned, operculate. Metraterm shorter than cirrus-sac, distal extremity with folded muscular pad. Vitellarium follicular, follicles sparse, reaching from pharynx to posterior extremity, almost confluent ventrally in forebody, confluent ventrally in post-testicular region; lateral and ventral to caeca.

Excretory pore dorsal, in mid post-testicular region; vesicle reaches to mid-region of anterior testis.

### Remarks

This species is characterised by molecular means (Table 1) and distinguished from similar congeners by the following morphological characteristics (Table 7). *Lepotrema clavatum* is larger, with a slightly longer forebody, a longer pre-oral lobe, a larger oral sucker, a shorter oesophagus, a longer intestinal bifurcation to ventral sucker distance, a much longer cirrus-sac, larger gonads, a shorter post-testicular distance and longer caeca. *Lepotrema acanthochromidis* n. sp. has a longer pre-oral lobe, the oesophagus tends to be longer, the intestinal bifurcation is further from the ventral sucker, the cirrus-sac is longer and post-testicular region is slightly shorter. *Lepotrema adlardi* is slightly narrower, particularly in the forebody, with a longer pre-oral lobe and prepharynx, a much longer pre-vitelline distance and a longer cirrus-sac and caeca. *Lepotrema amansis* n. sp. never gets as large, usually has a less distinct pre-oral lobe, wider suckers, a slightly longer pharynx, a longer intestinal bifurcation to ventral sucker distance, a slightly shorter pre-vitelline distance, a longer cirrus-sac and a shorter post-testicular distance. *Lepotrema cirripectis* n. sp. has a slightly shorter prepharynx and oesophagus and a smaller pharynx, the intestinal bifurcation is further from the ventral sucker and has a longer cirrus-sac, ventral sucker to ovary distance and caeca. *Lepotrema hemitaurichthydis* n. sp. has a slightly longer forebody, the intestinal bifurcation is further from the ventral sucker, the cirrus-sac is longer, the testes are larger, post-testicular region is shorter and the caeca are longer. *Lepotrema justinei* n. sp. is wider, with a longer pre-oral lobe, relatively slightly larger suckers, an indistinct prepharynx, a longer pharynx, a shorter oesophagus, a longer cirrus-sac, no distinct separation of ventral sucker and ovary and larger gonads. *Lepotrema melichthydis* n. sp. has a more distinct pre-oral lobe, a shorter prepharynx, a longer pharynx, a shorter oesophagus, the intestinal bifurcation is further from the ventral sucker, the pre-vitelline distance shorter and the cirrus-sac and caeca are longer. *Lepotrema moretonense* n. sp. has a more distinct pre-oral lobe, a shorter prepharynx, a longer intestinal bifurcation to ventral sucker distance, a larger oral sucker and pharynx, a longer cirrus-sac and a shorter post-testicular distance.

Bray et al. (1993) described and illustrated this form, based on two specimens. They stated that it was “similar to *L. clavatum* in many metrical features and possess a distinct folded muscular metraterm pad. They differ in the short cirrus-sac, just overlapping the ventral sucker, and the straight ejaculatory duct”.

Infections of this form have been found, all as single infections, in five of 41 *A. curacao* examined from off Heron Island, but in none of 30 examined from off Lizard Island. It has also been found in just one of nine *Amphiprion akindynos* examined at Heron Island. The species has not been detected in over 1,300 individuals of 40 other pomacentrid species examined in the region.

***Lepotrema canthescheniae*** **Bray & Cribb, 1996 emend.**

*Type-host*: *Cantheschenia grandisquamis* Hutchins (Tetradontiformes: Monacanthidae), large-scaled leatherjacket.

*Type-locality*: Off Heron Island, Great Barrier Reef, Australia.

*Prevalence*: In 2 of 42 fish examined.

### Remarks

In this species, along with only *L. xanthichthydis*, the vitellarium reaches to the oral sucker. These worms differ in cirrus-sac length, the lack or presence of uterine coils in the forebody, and egg-length (Table 6; see also Fig. 28).

The original description of *L. canthescheniae* was based on three specimens from two individual *C. grandisquamis* from off Heron Island (Bray & Cribb, 1996c). We have since examined nine more individuals of *C. grandisquamis* (especially to obtain material for sequencing) but all have been uninfected. Total prevalence now stands at two of 42. We suspect that this species is oioxenous to *C. grandisquamis*, but the low prevalence detected is puzzling. This species is clearly morphologically distinct from the only other species of *Lepotrema* we have found in a monacanthid, *L. amansis* n. sp.

***Lepotrema monile*** **Bray & Cribb, 1998**

Syn. *Lepocreadium* sp. from *Pomacentrus* cf. *wardi* of Bray et al. (1993)

*Type-host*: *Pomacentrus wardi* Whitley (Perciformes: Pomacentridae), Ward’s damsel.

*Type-locality*: Off Heron Island, Great Barrier Reef, Australia.

*New material*

*Hosts*: *Pomacentrus amboinensis* Bleeker, Ambon damsel; *Pomacentrus chrysurus* Cuvier, whitetail damsel; *Stegastes apicalis* (De Vis), Australian Gregory (all Pomacentridae).

*Localities*: Off Lizard Island (14°40′S, 145°28′E) (ex *P. amboinensis* and *P. chrysurus*); off Heron Island (ex *S. apicalis*).

*Voucher material*: Ex *P. amboinensis* (QM G237567); ex *P. chrysurus* (QM G237565–6); ex *S. apicalis* (QM G237568).

*Prevalence*: Ex *P. amboinensis* (in 1 of 54 fish examined); ex *P. chrysurus* (in 1 of 26 fish examined); ex *S. apicalis* (in 2 of 27 fish examined).

*Representative DNA sequences*: ITS2 rDNA, two identical replicates (one submitted to GenBank MH730009); *cox*1 mtDNA, two identical replicates (both submitted to GenBank MH730048–49); 28S rDNA, one sequence (submitted to GenBank MH730024).

### Remarks

**Figs. 29–35:**
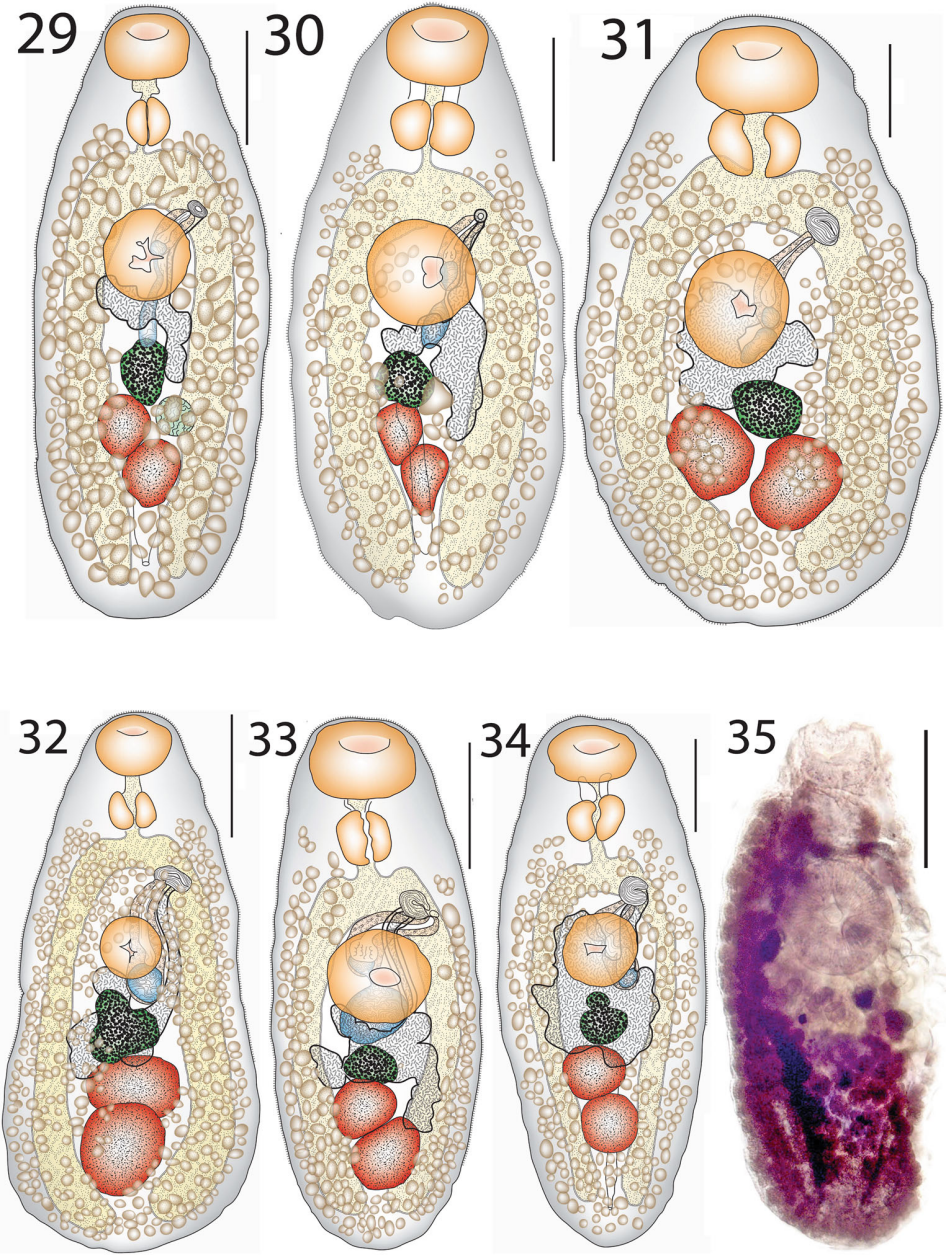
*Lepotrema* spp. 29, 30, *Lepotrema monile* Bray & Cribb, 1998. 29, ex *Pomacentrus wardi*, off Heron Island, ventral view (redrawn from Bray & Cribb, 1998); 30, ex *Stegastes apicalis*, off Heron Island, ventral view; 31, *Lepotrema* sp. 1 ex *Rhinecanthus aculeatus*, off Lizard Island, ventral view; 32, *Lepotrema* sp. 2 ex *Rhinecanthus aculeatus*, Palau, ventral view; 33, *Lepotrema* sp. 3 ex *Sufflamen chrysopterum*, off Heron Island, ventral view; 34, *Lepotrema* sp. 4 ex *Parma polylepis*, off Heron Island, ventral view; 35, *Lepotrema* sp. 5, micrograph of hologenophore ex *Ctenochaetus striatus*, off Heron Island, ventral view. *Scale-bars*: 200 μm

This species is distinct in that it lacks a prominent folded muscular pad at the distal metraterm, having a reduced muscular sphincter. It differs genetically from all other *Lepotrema* species for which molecular data are available. New measurements are given in Table 7; see also Figs. 29–30.

It also differs from other similar species in the following characteristics. *Lepotrema clavatum* is larger, with a longer forebody, a shorter pre-vitelline distance, a longer cirrus-sac, a shorter ventral sucker to ovary distance, slightly larger gonads, a shorter post-testicular region and longer caeca. *Lepotrema adlardi* is narrower and mostly larger, with a longer forebody, slightly smaller suckers, a distinctly longer prepharynx, a longer oesophagus and pre-vitelline distance, a shorter ventral sucker to ovary distance and a slightly smaller ovary. *Lepotrema acanthochromidis* n. sp. has a slightly longer intestinal bifurcation to ventral sucker distance, a longer cirrus-sac and a shorter ventral sucker to ovary distance. *Lepotrema amansis* n. sp. has a slightly longer forebody, larger suckers and pharynx, a longer intestinal bifurcation to ventral sucker distance, a shorter pre-vitelline distance, a longer cirrus-sac, a shorter ventral sucker to ovary distance and post-testicular region and possibly slightly shorter eggs. *Lepotrema amblyglyphidodonis* n. sp. has a less distinct pre-oral lobe and a shorter ventral sucker to ovary distance. *Lepotrema cirripectis* n. sp. has a less distinct pre-oral lobe, a longer intestinal bifurcation to ventral sucker distance and cirrus-sac and possibly slightly longer caeca. *Lepotrema hemitaurichthydis* n. sp. has a slightly longer forebody, slightly larger suckers, a longer intestinal bifurcation to ventral sucker distance, a longer cirrus-sac, shorter ventral sucker to ovary distance and post-testicular region, longer caeca and possibly slightly longer eggs. *Lepotrema justinei* n. sp. is broader, with a distinct pre-oral lobe, a shorter prepharynx, a larger pharynx, shorter pre-vitelline distance and ventral sucker to ovary distances, larger gonads and shorter caeca. *Lepotrema melichthydis* n. sp. has a longer pre-oral lobe, a shorter prepharynx, a longer pharynx and intestinal bifurcation to ventral sucker distance, a shorter pre-vitelline distance, a longer cirrus-sac, a shorter ventral sucker to ovary distance, a smaller ovary and possibly slightly shorter eggs. *Lepotrema moretonense* n. sp. has a shorter prepharynx, a larger pharynx, a longer cirrus-sac, a shorter ventral sucker to ovary distance, a larger posterior testis and a shorter post-testicular region.

We have collected *L. monile* in seven of 61 *P. wardi* examined at Heron Island (Bray et al., 1993; Bray & Cribb, 1998). It has only been detected in one of 17 *Stegastes apicalis* from off Heron Island, but in none of 74 individuals of 12 other species of *Pomacentrus* examined from off Heron Island. However, it has been found in *P. amboinensis* (see Sun et al., 2012) and *P. chrysurus* (once each) from off Lizard Island, in each case the identification is based of morphology only. This species is interpreted as predominantly stenoxenous to the genus *Pomacentrus*. The low recorded prevalence in two of the species of *Pomacentrus* suggests that this species might well be found in more species of *Pomacentrus* given sufficient sampling.

***Lepotrema*** **sp. 1**

*Host*: *Rhinecanthus aculeatus* (Linnaeus) (Tetradontiformes: Balistidae), white-banded triggerfish.

*Locality*: Off Lizard Island (14°40′S, 145°28′E), Great Barrier Reef, Australia.

*Site in host*: Intestine.

*Voucher material*: QM G237569.

### Remarks

One specimen is available. Measurements of the specimen are given in Table 8; see also Fig. 31. It is broadly oval with almost symmetrical testes. In its nearly symmetrical testes this specimen resembles *L. justinei* n. sp., but the folded muscular pad on the metraterm is far smaller. Dyer et al. (1988) reported *L. clavatum* from *R. aculeatus* from off Okinawa, Japan, but without any descriptive matter, meaning that it is not possible to speculate rationally on whether that form is the same as the Lizard Island form, the Palau form (see below) or distinct. We have examined 38 individuals of this fish from off Lizard Island and another 23 from other sites in the region, but apart from *Lepotrema* sp. 2 (see below), only the single infection has been detected.

**Table 8:** Dimensions of *Lepotrema* spp. innom.

Species	*Lepotrema* sp. 1	*Lepotrema* sp. 2	*Lepotrema* sp. 3	*Lepotrema* sp. 4
Host	*Rhinecanthus aculeatus*	*Rhinecanthus aculeatus*	*Sufflamen chrysopterus*	*Parma polylepis*
Locality	Lizard Island	Palau	Heron Island	Heron Island
n	1	2	1	1
Body	1,336 × 788	789–899 × 299–420	835 × 320	1,133 × 451
Forebody	499	317–336	342	425
Pre-oral lobe	23	4–13	0	19
Oral sucker	194 × 263	86–93 × 119–125	121 × 160	127 × 183
Prepharynx	0	32–34	19	21
Pharynx	141 × 202	61–64 × 65–79	85 × 84	91 × 118
Oesophagus	0	20–21	26	54
Intestinal bifurcation to ventral sucker	142	109–129	90	112
Pre-vitelline distance	270	168–169	168	255
Vitellarium to ventral sucker	229	149–167	174	170
Ventral sucker	292 × 322	95–106 × 99–111	148 × 158	159 × 159
Cirrus-sac	?	250–243 × 54–71	225 × ?	246 × 72
Ventral sucker to ovary	21	22–25	33	15
Ovary	128 × 152	80–114 × 113–93	58 × 83	119 × 101
Ovary to anterior testis	0	0	0	0
Anterior testis	201 × 204	103–110 × 130–151	103 × 103	117 × 129
Distance between testes	0	0	0	0
Posterior testis	210 × 196	110–136 × 132–137	116 × 109	128 × 130
Post-testicular distance	203	85–102	90	190
Post-caecal distance	82	27–34	55	84
Eggs	64 × 26	58–61 × 30–33	58 × 28	58 × 41
Width (%)^a^	59.0	37.9–46.7	38.3	39.8
Forebody (%)^a^	37.4	37.4–40.2	41.0	37.5
Sucker length ratio	1:1.51	1:1.10–1.14	1:1.22	1:1.26
Sucker width ratio	1:1.22	1:0.83–0.88	1:0.99	1:0.87
Oral sucker: pharynx width	1:1.30	1:1.59–1.82	1:1.90	1:1.55
Ventral sucker to ovary (%)^a^	1.58	2.73–2.74	3.95	1.31
Post-testicular distance (%)^a^	15.2	10.7–11.3	10.8	16.7
Prepharynx (%)^a^	0	3.59–4.36	2.28	1.85
Oesophagus (%)^a^	0	2.18–2.63	3.11	4.74
Intestinal bifurcation to ventral sucker distance (%)^a^	10.6	13.8–14.4	10.8	9.88
Vitellarium to ventral sucker distance	17.1	18.6–19.0	20.8	15.0
Ovary to anterior testis (%)^a^	0	0	0	0
Distance between testes (%)^a^	0	0	0	0
Cirrus-sac length (%)^a^	?	27.0–31.7	26.9	21.7
Pre-vitelline distance (%)^a^	20.2	18.8–21.3	20.1	22.5
Anterior testis length (%)^a^	15.1	12.3–13.1	12.3	10.3
Posterior testis length (%)^a^	15.7	14.0–15.2	13.9	11.3

^a^%, percent of body length

***Lepotrema*** **sp. 2**

*Host*: *Rhinecanthus aculeatus* (Linnaeus) (Tetraodontiformes: Balistidae), white-banded triggerfish.

*Locality*: Off Palau (07°30′N, 134°30′E).

*Site in host*: Intestine.

*Prevalence*: In 1 of 2 fish examined.

*Voucher material*: QM G237570–1.

### Remarks

Two specimens are available (Table 8; Fig. 32). They appear distinctly different from the specimen (*Lepotrema* sp. 1) reported from the same host at Lizard Island. One of two individuals of *R. aculeatus* examined from off Palau was infected.

***Lepotrema*** **sp. 3**

Syn. *Lepotrema clavatum* of Bray & Cribb (1996c) in part

*Host*: *Sufflamen chrysopterum* (Bloch & Schneider) (Tetraodontiformes: Balistidae), halfmoon triggerfish.

*Locality*: Off Heron Island (23°27′S, 151°55′E), Great Barrier Reef, Australia.

*Site in host*: Intestine.

*Prevalence*: In 1 of 67 fish examined.

*Voucher material*: QM G212867.

### Remarks

Only one specimen is available (Table 8; Fig. 33). We have now examined 67 individuals of *S. chrysopterum* at Heron Island with only one infection detected. This species is clearly different from *Lepotrema justinei* n. sp., the other form found in *Sufflamen.* It is a smaller, much narrow worm with almost tandem testes and the ovary is distinctly separated from the ventral sucker.

***Lepotrema*** **sp. 4**

Syn. *Lepotrema clavatum* of Bray et al. (1993) and Barker et al. (1994) in part

*Host*: *Parma polylepis* Günther (Perciformes: Pomacentridae), banded Parma.

*Locality*: Off Heron Island (23°27′S, 151°55′E), Great Barrier Reef, Australia.

*Site in host*: Intestine.

*Prevalence*: In 2 of 6 fish examined.

*Voucher material*: QM GL 14773–4; BM(NH) 1992.10.5.6.

### Remarks

Three adult specimens were collected, measurements of one are given in Table 8 (see also Fig. 34). This form was originally reported from two of six *P. polylepis* examined from off Heron Island. This pomacentrid is not common at the sites we have surveyed on the GBR, and we have not detected this fish there since 1992.

***Lepotrema*** **sp. 5**

*Host*: *Ctenochaetus striatus* (Quoy & Gaimard) (Perciformes: Acanthuridae), striated surgeonfish.

*Locality*: Off Heron Island (23°27′S, 151°55′E), Great Barrier Reef, Australia.

*Site in host*: Intestine.

*Prevalence*: In 1 of 42 fish examined.

*Voucher material*: Hologenophore QM G237572.

*Representative DNA sequences*: ITS2 rDNA, one sequence (submitted to GenBank MH730010); *cox*1 mtDNA, one sequence (submitted to GenBank MH730050); 28S rDNA, one sequence (submitted to GenBank MH730022).

### Remarks

One *Lepotrema* specimen was found in 119 specimens of this host, 42 of which were from off Heron Island. This specimen (Fig. 35) has a relatively exceptionally large ventral sucker which appears to distinguish it from all other recognised species of *Lepotrema*. It also clearly distinct from all the species for which molecular data exists on the basis of ITS2 and *cox*1 data. In the 28S phylogram (Fig. 1B) this form is sister to *Lepotrema amblyglyphidodonis* n. sp., but this relationship has poor support.

***Lepotrema incisum*** **(Hanson, 1955) Bray & Cribb, 1996**

Syn. *Lepocreadium incisum* Hanson, 1955

*Type-host*: *Melichthys niger* (Bloch) (as *buniva*) (Tetraodontiformes: Balistidae), black triggerfish.

*Type-locality*: Off Hawaii.

Remarks

**Figs. 36–39:**
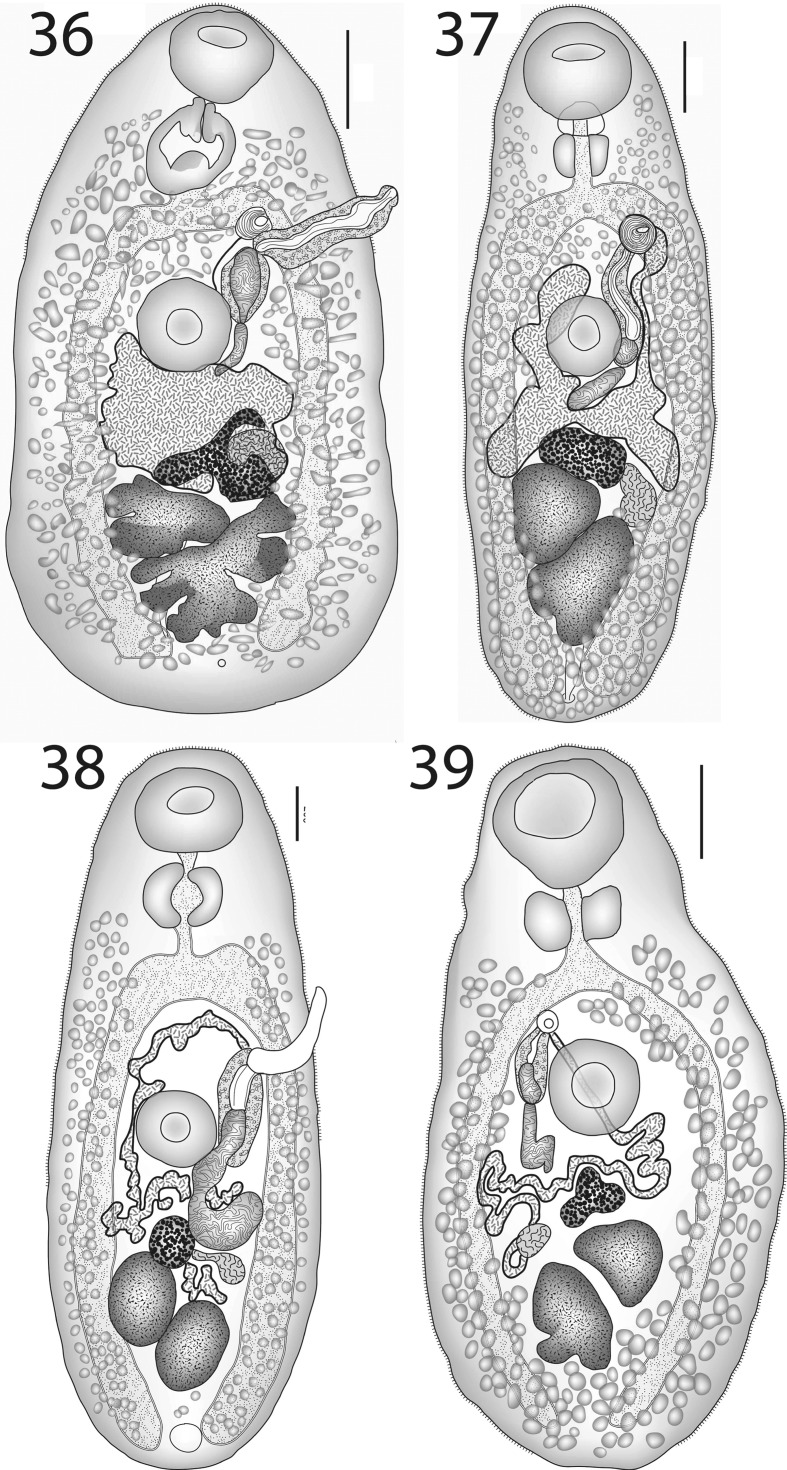
*Lepotrema* spp. 36, *Lepotrema incisum* (Hanson, 1955) ex *Melichthys buniva*, Hawaii, ventral view (redrawn from Hanson, 1955); 37, *Lepotrema xanthichthydis* (Yamaguti, 1970) ex *Xanthichthys ringens*, Hawaii, ventral view (redrawn from Yamaguti, 1970); 38, *Lepotrema cylindricum* (Wang, 1989) n. comb. ex *Monacanthus chinensis* or *Navodon septentrionalis*, off Fujian, China, ventral view (redrawn from Wang, 1989); 39, *Lepotrema navodonis* (Shen, 1986) n. comb. ex *Thamnoconus modestus*, off Zhejiang, China, ventral view (redrawn from Shen, 1986). *Scale-bars*: 200 μm

Important differentiating characters include the shape, width and the deeply incised testes (Table 9; Fig. 36). The host-species was quoted by Hanson (1955) as *Melichthys buniva*, but according to Randall (2007) this is a synonym of an Atlantic species, and is a misidentification of *M. niger*.

**Table 9:** Dimensions of *Lepotrema incisum* (Hanson, 1955), *L. xanthichthydis* (Yamaguti, 1970), *L. cylindricum* (Wang, 1989) and *L. navodonis* (Shen, 1986) derived from literature sources

Species	*Lepotrema incisum*	*Lepotrema xanthichthydis*	*Lepotrema cylindricum*	*Lepotrema navodonis*
Host	*Melichthys buniva*	*Xanthichthys ringens*	*Monacanthus chinensis* & *Navodon septentrionalis*	*Thamnaconus modestus*
Locality	Hawaii	Hawaii	Fujian, China	Zhejiang, China
Source	Hanson (1955)	Yamaguti (1970)	Wang (1989)	Shen (1986)
Body	1,132–1,432 × 501–785	1,200–1,760 (2,072^a^) × 520–700	2,600–2,920 × 1,040–1,120	1,411–1,751 × 612–799
Forebody	553^a^	873^a^	1,254^a^	582^a^
Pre-oral lobe	0^a^	45^a^	68^a^	29^a^
Oral sucker	169–193 × 200–223	120–270 × 190–310	312–314 × 300–400	170–323 × 272–323
Prepharynx	15–46	50–60	48^a^	71–84
Pharynx	131–177 × 154–177	100–130 × 90–170	232 × 286^a^	85–153 × 187–272
Oesophagus	23–32	40–90	109^a^	34–68
Intestinal bifurcation to ventral sucker	147^a^	309^a^	505^a^	156^a^
Pre-vitelline distance	21^a^	291^a^	599^a^	361^a^
Vitellarium to ventral sucker	335^a^	582^a^	655^a^	221^a^
Ventral sucker	177–193 × 177–200	170–230 × 170–230	273 × 300^a^	204–255 × 204–244
Cirrus-sac	108–185 × 77–93	200–360 × 50–80	436 × 166^a^	221–323 × 68–85
Ventral sucker to ovary	13^a^	145^a^	184^a^	61^a^
Ovary	200 × 280^a^	90–210 × 140–260	191 × 170^a^	102–238 × 153–204
Ovary to anterior testis	0	0^a^	0^a^	29^a^
Anterior testis	162–216 × 146–269	160–370 × 200–230	348 × 245^a^	153–289 × 186–289
Distance between testes	0	0^a^	0^a^	25^a^
Posterior testis	169–239 × 162–354	160–370 × 200–230	327 × 225^a^	170–255 × 187–204
Post-testicular distance	135^a^	218^a^	280^a^	205^a^
Post-caecal distance	100^a^	73^a^	55^a^	66^a^
Eggs	38–46 × 23–30	46–56 × 25–33	56–60 × 35–42	48–54 × 24–27
Width (%)^b^	44.3–54.8	39.8–43.3	38.4–40.0	43.4–45.6
Forebody (%)^b^	38.8^a^	42.1^a^	47.4^a^	37.8^a^
Sucker length ratio	1:1.00–1.05	1:0.85–1.42	1:0.87^a^	1:0.79–1.20
Sucker width ratio	1:08.9–0.90	1:0.74–0.89	1:0.76^a^	1:0.75–0.79
Oral sucker: pharynx width	1:1.26–1.30	1:1.82–2.11	1:1.38^a^	1:1.19–1.45
Ventral sucker to ovary (%)^b^	0.9^a^	7.00^a^	6.96^a^	3.96^a^
Post-testicular distance (%)^b^	9.5^a^	10.5^a^	10.6^a^	13.3^a^
Prepharynx (%)^b^	1.3–3.2	3.41–4.17	1.81^a^	4.80–5.03
Oesophagus (%)^b^	2.0–2.2	3.33–5.11	4.12^a^	2.41–3.88
Intestinal bifurcation to ventral sucker distance (%)^b^	10.3^a^	14.9^a^	19.1^a^	10.1^a^
Vitellarium to ventral sucker distance	23.5^a^	28.1^a^	24.8^a^	14.3^a^
Ovary to anterior testis (%)^b^	0	0^a^	0^a^	1.88^a^
Distance between testes (%)^b^	0	0^a^	0^a^	1.62^a^
Cirrus-sac length (%)^b^	9.5–12.9	16.7–20.4	16.5^a^	15.7–18.4
Pre-vitelline distance (%)^b^	15.3^a^	14.0^a^	22.6^a^	22.4^a^
Anterior testis length (%)^b^	14.3–15.1	13.3–21.0	13.2^a^	10.8–16.5
Posterior testis length (%)^b^	14.9–16.7	13.3–21.0	12.4^a^	12.0–14.6

^a^From the illustration; ^b^%, percent of body length

***Lepotrema xanthichthydis*** **(Yamaguti, 1970**) **Bray & Cribb, 1996**

Syn. *Lepocreadium xanthichthydis* Yamaguti, 1970

*Type-host*: *Xanthichthys ringens* (Linnaeus) (Tetraodontiformes: Balistidae), sargassum triggerfish.

*Type-locality*: Off Hawaii.

### Remarks

Important differentiating characters include the forebody length, the anterior position of the bifurcation and the vitelline extent, the cirrus-sac not reaching into the hindbody, the uterine coil in the forebody and the egg length (46–56) (Yamaguti, 1970) (Table 9; Fig. 37).

***Lepotrema cylindricum*** **(Wang, 1989) n. comb.**

Syn. *Preptetos cylindricus* Wang, 1989

*Type-host*: *Thamnaconus septentrionalis* (Günther) (first host listed) (Tetraodontiformes: Monacanthidae), drab leatherjacket.

*Other host*: *Monacanthus chinensis* (Osbeck) (Monacanthidae), fan-bellied leatherjacket.

*Type-locality*: Off Pingtan County, Fujian, China.

### Remarks

Although erected in the genus *Preptetos*, this species is consistent with *Lepotrema* and we formally propose the new combination here. It is much bigger than any other described species of *Lepotrema* (see Wang, 1989) (Table 9; Fig. 38). We note that we have examined 23 specimens of *Monacanthus chinensis* from Moreton Bay without finding this or any other species of *Lepotrema*.

***Lepotrema navodonis*** **(Shen, 1986) n. comb.**

Syn. *Lepocreadium navodoni* Shen, 1986

*Type-host*: *Thamnaconus modestus* (Günther) (Tetraodontiformes: Monacanthidae), Korean black scraper.

*Type-locality*: Off Nongbo, Zhejiang, China.

### Remarks

Although erected in the genus *Lepocreadium*, this species is consistent with *Lepotrema* and we formally propose the new combination here. It appears to be of an unusual pyriform shape with the narrower part anterior and the cirrus-sac beside the ventral sucker (Table 9; Fig. 39). In the original paper it is compared with *L. clavatum* and *L. xanthichthydis*, both considered in *Lepocreadium* by Shen (1986), but considered *Lepotrema* here. The cirrus-sac is described as to the right of the ventral sucker, an unusual feature for *Lepotrema*. It is noteworthy that *T. modestus* has also been reported as a host of *L. clavatum*. It seems possible that either *L. navodonis* could be a synonym of *L. clavatum*, or that *L. clavatum* may not really infect *T. modestus*.

**Phylogenetic results**

Neighbour-joining phylograms produced for the ITS2 and *cox*1 datasets demonstrate the complete lack of intraspecific variation in ITS2 rDNA (except for *L. hemitaurichthydis*) and its frequency among *cox*1 sequences (6 of 9 species for which at least two sequences were obtained). The topologies from the two analyses were substanially different (Figs. 1A and 2, respectively), with only one relationship shared between the two analyses; *L*. *hemitaurichthydis* n. sp. and *L*. *moretonense* n. sp. were sister taxa in analyses for both regions. Due to the high level of difference between the two phylograms, interpretation of relationships within the genus are instead based on the partial 28S rDNA dataset; this region has been shown to be more reliable for inference of phylogenetic relationships (Blasco-Costa et al., 2016), and has been used for inferring relationships within the Lepocreadiidae in several studies (Bray et al., 2009b; Bray & Cribb, 2012; Bray et al., 2018). Similar to the ITS2 dataset, the 28S dataset contained few variable base positions, with two clearly distinct species (*L*. *cirripectis* n. sp. and *L*. *monile*) having identical sequences in the final dataset; they differ by a single indel in the partial 28S sequence alignment. Preliminary analysis that included all available lepocreadoid taxa showed that all *Lepotrema* species sequenced formed a single well-supported clade. Thus, due to the limited number of differences in the *Lepotrema* dataset, a reduced analysis relative to three closely related genera was conducted to limit the loss of informative characters. The phylogram produced by Bayesian inference analysis of this dataset is shown in Fig. 1B. *Lepotrema* as a genus was again well-supported, as was a clade containing all *Lepotrema* species to the exclusion of *L*. *melichthydis* n. sp. *Lepotrema acanthochromidis* n. sp., *L*. *hemitaurichthydis* n. sp. and *L*. *moretonense* n. sp. formed a stongly-supported clade, sister to a clade consisting of *L*. *amblyglyphidodonis* n. sp. and *Lepotrema* sp. 5. These five species formed a strongly-supported clade, sister to the poorly-supported clade of *L*. *adlardi*, *L*. *amansis* n. sp., *L*. *cirripectis* n. sp. and *L*. *monile*.

## Discussion

Recognition of species

We have taken an integrative approach to the recognition of species here. By this we mean that we have been influenced by evidence from morphology, genetics and host-specificity. Overall, we have found morphology to be inadequate for the convincing recognition of many species. Clear exceptions are *L. adlardi* (characterised by its body shape and exceptionally long prepharynx) and *L. amansis* n. sp. (characterised by a highly distinctive oral sucker). For almost all the other forms, distinction is not immediately obvious and is critically dependent on the capacity to examine multiple specimens and on corroborative indications from molecular data and host-specificity. In this respect, many of the species can be considered ‘cryptic’ in the broad sense of the term, if not in the strictest sense.

With the exception of the forms associated with species of *Hemitaurichthys* (a special case discussed separately below), all distinct genotypes in the ITS2 and clades in *cox*1 analyses were ultimately interpreted as relating to distinct species. The distinctions in ITS2 rDNA sequences were often small (as low as 1 bp), but the distinctions always correlated with morphological and host distributional distinctions. In addition, several of the combinations were in sympatry (removing the complexity of geographical distinction, see below). Thus, four taxa (*L. adlardi*, *L. amansis* n. sp., *L. cirripectis* n. sp. and *L. monile*) that all occur on the GBR, each differ from one another by only 2 bp in the ITS2 region. Differences in the 28S data for *L. amansis* n. sp., *L. cirripectis* n. sp. and *L. monile* were also low (0–2 bp); the two genotypes that were identical in the final dataset did differ by a single indel. However, these differences are entirely consistent with and supported by greater differences in the *cox*1 dataset (40–56 bp differences for the same four species). We thus have no hesitation in considering these small ITS2 and 28S differences as informative.

The final component of our integrated analysis was the nature of the host-specificity in the system. In general, the species recognised here were found consistently in just one fish species (oioxenous specificity) or in multiple congeners (*L. cirripectis* n. sp., *L. monile* and *L. moretonense* n. sp.) (stenoxenous specificity). This pattern is consistent with the overall pattern of trematode specificity in coral reef fishes recognised by Miller et al. (2011). Notably, Miller et al. (2011) mentioned *Lepotrema clavatum* as one of only four trematode species reported from multiple orders of GBR fishes. The doubt about the breadth of host-specificity of that species expressed in that paper has been supported here. The restriction of many *Lepotrema* species is quite remarkable given the context of the examination (reported for species of *Lepotrema* above) of often large numbers of closely-related fish species. Thus, four species are recognised in Pomacentridae; *L. adlardi* is found in only one species of *Abudefduf*, *L. acanthochromidis* n. sp. in only the single species of *Acanthochromis*, *L. monile* in only three species of *Pomacentrus* and *Stegastes apicalis*, and *L. amblyglyphidodonis* n. sp. only in single species each of *Amblyglyphidodon* and *Amphiprion*. This pattern of host-specificity leads to the suspicion that the form reported rarely from *Parma polylepis* may represent a further undescribed species. The pattern of oioxenous or stenoxenous host-specificity is not perfect. The clear example is that of *L. moretonense* n. sp. which occurs frequently in two species of *Prionurus* (Acanthuridae) and rarely (one adult, one immature) in *Selenotoca multifasciata*. Such rarities serve to emphasise the importance of finding multiple specimens and infected hosts to allow confidence that infections are not uninformative “stragglers”. The rarity of infections, such as the single specimens detected in one of 67 *Sufflamen chrysopterus* and one of 42 *Ctenochaetus striatus* at Heron Island, may well be an indication that the species concerned typically infect another fish species. However, the single specimen of *Lepotrema* from *C. striatus* is genetically unique, so it may be simply rare or localised in that host and the same may apply to the form from *S. chrysopterum.*

In our integrated approach to species recognition we have been unable to make much use of geographical considerations. Most of the species are known from just one site, or perhaps two sites within the GBR. Indeed, Cribb et al. (2016) observed that such limited reporting of species is general for the fish trematode fauna of the Indo-Pacific. A handful of species of *Lepotrema* have been reported from multiple localities. Specimens of *L*. *cirripectis* n. sp. from off Heron and Lizard Islands had identical ITS2 sequences and minor variation in *cox*1 sequences; this *cox*1 variation was at a level much lower than between clearly distinct species. *Lepotrema adlardi* has also been reported from the northern and southern GBR and off Western Australia, although it has only been sequenced from the southern GBR. More interestingly, *H. melichthydis* n. sp. is here reported from both Palau and the southern GBR, in the same fish species; slight morphological differences are interpreted as intraspecific variation. The most intriguing case of apparent widespread distribution is that of *L. hemitaurichthydis* n. sp. which is here reported from the off Australs and Marquesas Archipelagos in French Polynesia and from off Palau. In this case one host species is the same, the morphology is similar (although with some possible distinctions), and the ITS2 and *cox*1 sequences differ by 1 bp (in addition to a single indel) and 14 bp, respectively. Importantly, the intraspecific *cox*1 variation is greater than that for any ‘good’ species whereas it is considerably less than the interspecific variation between any combination of recognised species. In the face of this somewhat conflicting information, we propose a conservative approach, interpreting the forms from *H. polylepis* as a single species that demonstrates geographical genetic variation. This approach (interpreting low-level genetic differences in worms from the same or very similar hosts over geographic range as intra-specific variation) has been adopted for several trematode taxa of late [Cryptogonimidae: Miller et al. (2010b); Faustulidae: Diaz et al. (2013); Fellodistomidae: Downie et al. (2011); Monorchiidae: McNamara et al. (2014); Transversotrematidae: Cutmore et al. (2016)] although of these, only the study of the Monorchiidae incorporated both ITS2 and *cox*1 sequence data. Ultimately, we think that we do not yet know enough about the nature of the distribution of trematodes in the Indo-Pacific to be able to reliably interpret circumstances such as these.

Identification of the true host and geographical distribution of the type-species of *Lepotrema*, *L. clavatum*, remains important. On the basis of the patterns of host-specificity reported here, we think it unlikely that any species of the Balistidae, Chaetodontidae, Paralichthyidae, Pomacanthidae and Pomacentridae will prove to be typical hosts of this species. However, this does remain to be demonstrated and it should not be considered a *fait accompli*. As shown by Wee et al. (2017), host specificity of species of a single genus may vary quite dramatically and without evident explanation. A special problem with *L. clavatum* relating to the “type-series” is that these worms were flattened, whereas none of the material we collected was treated in this way. Certainly we advocate that molecular data should be a significant part of the argument when the overall status of *L. clavatum* receives further attention.

Significance of host specificity

Three families dominate the host records of *Lepotrema* spp. Using the number of records as an indicator it can be seen that 28% are from the Pomacentridae, 27% from the Balistidae and 23% from the Monacanthidae. Other reef fish families such as the Chaetodontidae (8%), Acanthuridae (3%) and Pomacanthidae (1.7%) are also represented, and the Blenniidae, which occurs frequently on coral reefs, has 5% of records. Records in the Paralichthyidae (1.7%) and the Scatophagidae (1.7%) indicate that the genus is not solely reef-associated. Nevertheless, it is clearly mostly associated with fishes on coral reefs. Two orders predominate with the Tetraodontiformes (50% of records) and the Perciformes (with 48%). The single report from a pleuronectiform appears anomolous and may be accidental, although the worms are described as ovigerous, but relatively small (Yamaguti, 1934).

This host distribution can best be considered in the context of the recent report that metacercariae of *Lepotrema clavatum* infect medusae of several cnidarian species in Japanese waters (Kondo et al., 2016). These authors further demonstrated that *Thamnaconus modestus* and *Psenopsis anomala*, which both live in association with jellyfish as juveniles, had both nematocysts and juvenile *L. clavatum* in their guts. In combination these data establish convincing evidence for at least one mode of transmission of this species. In this context it is noteworthy that Miyajima et al. (2011) demonstrated that *S. cirrhifer*, the type-host of *L. clavatum*, will feed willingly on medusae in captivity. Unfortunately, it is not obvious that feeding on medusae explains the distribution of other *Lepotrema* species. Although it is generally acknowledged that reports of ingestion of medusae by fishes are inadequate, of the fish reported as doing so in the reviews of Arai (2005) and Ates (1988), just two, *Melichthys niger* and *Xanthichthys ringens*, are known hosts for species of *Lepotrema*. For some of the remaining fish reported as hosts here, it seems plausible that medusae may be featured in their diet. Species of *Abudefduf*, *Amblyglyphidodon*, *Hemitaurichthys* and *Prionurus* all often feed in mid-water, although it is unclear whether medusae are, in fact, an important part of their diet. In contrast, species of *Amanses*, *Cantheschenia*, *Cirripectes*, *Pomacentrus*, *Rhinecanthus* and *Sufflamen* are typically demersal feeders that would not be expected to feed on medusae consistently. In this context it is noteworthy that *Amanses scopas* is an obligate coral feeder (Bacchet et al., 2006); Ward (pers. comm.) has identified fragments of the corals *Acropora cytherea* (Dana) and *Isopora* sp. from the intestine of specimens of *A. scopas* collected from off Heron Island. It seems possible, therefore, that species of this genus may have a predilection for cnidarians, both polyps and medusae, as second intermediate hosts. Regardless of the range of second intermediate hosts for species of this genus, the apparent randomness of distribution among apparently comparable fishes remains baffling.

**Host-parasite list**

Order Perciformes

Family Acanthuridae

*Prionurus maculatus* Ogilby

*Lepotrema moretonense* n. sp.

*Prionurus microlepidotus* Lacépède

*Lepotrema moretonense* n. sp.

*Ctenochaetus striatus* (Quoy & Gaimard)

*Lepotrema* sp. 5

Family Blenniidae

*Cirripectes filamentosus* (Alleyne & Macleay)

*Lepotrema cirripectis* n. sp.

*Cirripectes chelomatus* Williams & Maugé

*Lepotrema cirripectis* n. sp.

*Cirripectes stigmaticus* Strasburg & Schultz

*Lepotrema cirripectis* n. sp.

Family Chaetodontidae

*Hemitaurichthys polylepis* (Bleeker)

*Lepotrema hemitaurichthydis* n. sp.

*Hemitaurichthys thompsoni* Fowler

*Lepotrema hemitaurichthydis* n. sp.

*Hemitaurichthys zoster* (Bennett)

*Lepotrema clavatum* (*s.l.*)

Family Pomacanthidae

*Genicanthus semifasciatus* (Kamohara)

*Lepotrema clavatum* (*s.l.*)

Family Pomacentridae

*Abudefduf bengalensis* (Bloch)

*Lepotrema adlardi* (Bray, Cribb & Barker, 1993)

*Acanthochromis polyacanthus* (Bleeker)

*Lepotrema acanthochromidis* n. sp.

*Amblyglyphidodon curacao* (Bloch)

*Lepotrema amblyglyphidodonis* n. sp.

*Amphiprion akindynos*

*Lepotrema amblyglyphidodonis* n. sp.

*Dascyllus albisella* Gill

*Lepotrema clavatum* (*s.l.*)

*Parma polylepis* Günther

*Lepotrema* sp. 4

*Pomacentrus amboinensis* Bleeker

*Lepotrema monile* Bray & Cribb, 1998

*Pomacentrus chrysurus* Cuvier

*Lepotrema monile* Bray & Cribb, 1998

*Pomacentrus wardi* Whitley

*Lepotrema monile* Bray & Cribb, 1998

*Stegastes apicalis* (De Vis)

*Lepotrema monile* Bray & Cribb, 1998

Family Scatophagidae

*Selenotoca multifasciata* (Richardson)

*Lepotrema moretonense* n. sp.

Order Pleuronectiformes

Family Paralichthyidae

*Pseudorhombus cinnamoneus* (Temminck & Schlegel)

*Lepotrema clavatum* (*s.l.*)

Order Tetraodontiformes

Family Balistidae

*Melichthys niger* (Bloch)

*Lepotrema clavatum* (*s.l.*)

*Lepotrema incisum* (Hanson, 1955)

*Melichthys vidua* (Richardson)

*Lepotrema clavatum* (*s.l.*)

*Lepotrema melichthydis* n. sp.

*Odonus niger* (Rüppell)

*Lepotrema clavatum* (*s.l.*)

*Rhinecanthus aculeatus* (Linnaeus)

*Lepotrema clavatum* (*s.l.*)

*Lepotrema* sp. 1

*Lepotrema* sp. 2

*Sufflamen chrysopterum* (Bloch & Schneider)

*Lepotrema* sp. 3

*Sufflamen fraenatum* (Latreille)

*Lepotrema justinei* n. sp.

*Xanthichthys ringens* (Linnaeus)

*Lepotrema xanthichthydis* (Yamaguti, 1970)

Family Monacanthidae

*Amanses scopas* (Cuvier)

*Lepotrema amansis* n. sp.

*Cantheschenia grandisquamis* Hutchins

*Lepotrema canthescheniae* Bray & Cribb, 1996

*Monacanthus chinensis* (Osbeck)

*Lepotrema cylindricum* (Wang, 1989) n. comb.

*Stephanolepis cirrhifer* (Temminck & Schlegel)

*Lepotrema clavatum* (*s.s.*)

*Thamnaconus modestus* (Günther)

*Lepotrema clavatum* (*s.l.*)

*Lepotrema navodonis* (Shen, 1986) n. comb.

*Thamnaconus septentrionalis* (Günther)

*Lepotrema cylindricum* (Wang, 1989) n. comb.
